# Cellular Origins and Context-Dependent Prognostic Effects of Lactate Metabolism Genes Reveal Novel Molecular Subtypes in Gastric Cancer

**DOI:** 10.3390/cimb48050477

**Published:** 2026-05-04

**Authors:** Xiaoxuan Pan, Xin Chen, Chunyuan Zhang, Yongtong Huan, Jieru Han

**Affiliations:** 1 Second Clinical Medical College, Heilongjiang University of Chinese Medicine, Harbin 150040, China; panxiaoxuan2005@126.com (X.P.); huanyongtong@outlook.com (Y.H.); 2 First Clinical Medical College, Heilongjiang University of Chinese Medicine, Harbin 150040, China; radinen@outlook.com (X.C.); 18009333891@163.com (C.Z.); 3 School of Basic Medical Sciences, Heilongjiang University of Chinese Medicine, Harbin 150040, China

**Keywords:** GC, lactate metabolism, molecular subtyping, prognostic model, *FABP4*, tumor microenvironment, external validation

## Abstract

Objective: Gastric cancer (GC) exhibits profound heterogeneity, yet the contribution of lactate metabolism reprogramming to this diversity and its cellular basis remain incompletely understood. This study aimed to dissect GC heterogeneity through lactate metabolism-related genes (LMRGs), with a focus on the cellular origins and context-specific functions of key genes. Methods: We performed consensus clustering of TCGA GC samples (*n* = 375) using a curated set of 49 LMRGs. A multi-step screening strategy was employed to identify hub genes. Single-cell RNA-seq data were integrated to map the cellular sources of key genes. Subtype-specific analyses of mutation, expression, and prognosis were conducted. A prognostic model was constructed, but its cross-platform generalizability was critically evaluated to explore the functional heterogeneity of its constituent genes. Results: We identified two distinct GC subtypes: G1, a glycolytic and immunosuppressive subtype associated with poor prognosis, and G2, an immune-activated subtype with better prognosis. Crucially, single-cell analysis revealed that the hub gene *HK2* is enriched in NK cells and pDCs, while *FABP4* exhibits a dual cellular origin, being expressed in both proliferative CD8+ T cells and fibroblasts. This dual origin provides a mechanistic basis for the gene’s context-dependent behavior: while *FABP4* appeared protective in overall models, it acted as a significant risk factor within the G2 subtype (HR = 1.71, *p* = 0.017) and in an external validation cohort (HR = 2.64). A derived prognostic model failed external cross-platform validation, a phenomenon driven by the reversal of risk effects for *FABP4* and other genes across different populations. Conclusions: This study uncovers two distinct metabolic-immune subtypes of GC and demonstrates that the prognostic effect of *FABP4* is not fixed but is highly dependent on its cellular source and the tumor microenvironmental context. Our findings generate a testable hypothesis regarding *FABP4*’s role in balancing anti-tumor immunity and stromal promotion. More broadly, the failure of our cross-platform model serves as a cautionary tale on the limitations of single-cohort-derived signatures and underscores the necessity of integrating single-cell resolution to unravel the biological complexity underlying prognostic biomarkers.

## 1. Introduction

GC is one of the most common malignancies worldwide, with approximately 1.089 million new cases and 768,000 deaths globally in 2020, ranking fifth in incidence and fourth in mortality among all malignant tumors, respectively [1]. Despite continuous advancements in diagnostic and therapeutic techniques, the pronounced heterogeneity of GC leads to significant variability in patient prognosis. The traditional TNM staging system struggles to fully capture the complex biological behavior of the tumor [2]. In recent years, tumor metabolic reprogramming has been recognized as a key factor driving tumor heterogeneity, characterized by lactate accumulation resulting from aerobic glycolysis (i.e., the “Warburg effect”) [3,4]. Lactate has transformed from a mere metabolic waste product into a multifunctional signaling molecule and substrate, profoundly shaping the heterogeneity of the tumor microenvironment by mediating metabolic symbiosis, immune evasion, and epigenetic modifications (e.g., lactylation) [5]. Therefore, abnormal lactate metabolism has emerged as a new hallmark of cancer, and the expression patterns of its related genes are expected to reveal the internal state of tumors, serving as a potential basis for molecular subtyping [6,7].

Currently, although some studies have constructed prognostic models for GC based on LMRGs, most have focused on the predictive performance of the models, with insufficient exploration of the complexity of gene functions and their heterogeneous roles across different molecular subtypes [8]. Differences in the cellular origin between tumor cells and immune or stromal cells lead to variations in the expression of key genes, which in turn exhibit opposing functions, and their prognostic effects may reverse depending on changes in the subtype context [9]. This heterogeneity in gene function, rooted in cell type and subtype background, is a crucial manifestation of tumor complexity and represents key information that needs to be deeply explored for precise subtyping strategies [10].

Based on this, the present study utilized transcriptomic data of GC from TCGA to systematically integrate lactate metabolism-related gene sets and identify novel molecular subtypes of GC through consensus clustering [11]. Subsequently, differential expression analysis, survival analysis, and PPI networks were employed to screen hub genes. Furthermore, single-cell transcriptomic data were integrated to elucidate the cellular origins of key genes, and their associations with mutation status, expression levels, and prognosis were deeply investigated within specific subtypes [12]. This study aims to advance from “subtype identification” to “subtype-specific analysis,” revealing the complexity of lactate metabolism-related gene functions and providing a novel heterogeneity-based perspective for precision subtyping and targeted strategies in GC.

## 2. Materials and Methods

### 2.1. Data Acquisition and Preprocessing

The GC transcriptomic data and corresponding clinical information used in this study were downloaded from TCGA database (https://portal.gdc.cancer.gov/ accessed on 10 February 2026). We extracted the STAR-Counts data and converted them to Transcripts Per Million (TPM) format, followed by log2(TPM + 1) transformation. Ultimately, a total of 375 GC samples with both RNA-seq data and complete clinical follow-up information were included for subsequent analyses.

### 2.2. Identification of Molecular Subtypes of Gastric Cancer

To identify potential molecular subtypes of GC, we performed consensus clustering analysis on the 375 samples using the R package ConsensusClusterPlus (version 1.54.0). The parameters were set as follows: maximum evaluated cluster number maxK = 6, number of resampling iterations reps = 100, proportion of items sampled per iteration pItem = 0.8, clustering algorithm clusterAlg = “hc”, and inner linkage method innerLinkage = “ward.D2”. Based on the cumulative distribution function (CDF) curve, the CDF Delta area, and the consensus matrix heatmap, the optimal number of clusters was determined to be k = 2. The two subtypes were designated as G1 (*n* = 160) and G2 (*n* = 215), respectively. The R package pheatmap (version 1.0.12) was used to visualize the differentially expressed genes (DEGs) between the subtypes.

### 2.3. Compilation of Lactate Metabolism-Related Gene Set

To systematically capture the multifaceted biological processes of lactate metabolism, we compiled a comprehensive gene set of 49 LMRGs using a three-tiered prioritization strategy. The selection criteria were designed to establish a robust foundation for subsequent molecular subtyping, ensuring both breadth of coverage and biological specificity:

Tier 1: Core Canonical Pathways (High Priority). Genes directly involved in the core biochemical reactions of glycolysis, gluconeogenesis, and pyruvate metabolism were extracted from the KEGG pathway database (hsa00010: Glycolysis/Gluconeogenesis; hsa00620: Pyruvate metabolism) and the Reactome pathway database (R-HSA-70326: Glucose metabolism). These genes constitute the essential enzymatic machinery of lactate production and consumption.

Tier 2: Essential Transporters and Regulatory Kinases (Medium Priority). We expanded the set to include key solute carriers responsible for lactate and glucose transport across cellular membranes (e.g., the SLC16A monocarboxylate transporter family and the SLC2A glucose transporter family), as well as critical regulatory kinases such as the pyruvate dehydrogenase kinase (PDK) family. These components are indispensable for metabolic flux control and intercellular lactate shuttling.

Tier 3: Literature-Derived Emerging Regulators (Supplementary Priority). To ensure our gene set reflects the latest advances in the field, we curated additional genes from recent peer-reviewed literature that have been reported to play novel or context-specific roles in tumor lactate metabolism and microenvironmental remodeling (e.g., *MCTP1*, *MCTP2*).

The complete list of the 49 LMRGs, categorized by their functional classification and source prioritization, is provided in Table 1. This structured compilation serves as the initial, biologically grounded input for the subsequent consensus clustering and screening steps.

### 2.4. Differential Expression and Functional Enrichment Analysis

DEGs between the G1 and G2 subtypes were screened using the R package Limma (version 3.40.2). Multiple testing correction was performed using the Benjamini–Hochberg (BH) method to control the false discovery rate (FDR), as implemented in the topTable function with the default adjust.method = “BH” parameter. The screening threshold was set to |log2FC| > 1 and adjusted *p*-value (FDR) < 0.05. Differential genes were visualized using volcano plots and heatmaps.

Subsequently, Gene Ontology (GO) and Kyoto Encyclopedia of Genes and Genomes (KEGG) enrichment analyses of the differentially expressed genes were performed using the R package clusterProfiler (version 3.18.0) to explore their potential biological functions. The significance threshold for enrichment was set at adjusted *p* < 0.05.

### 2.5. Preliminary Screening of Prognostic-Related Genes

**Figure 1:**
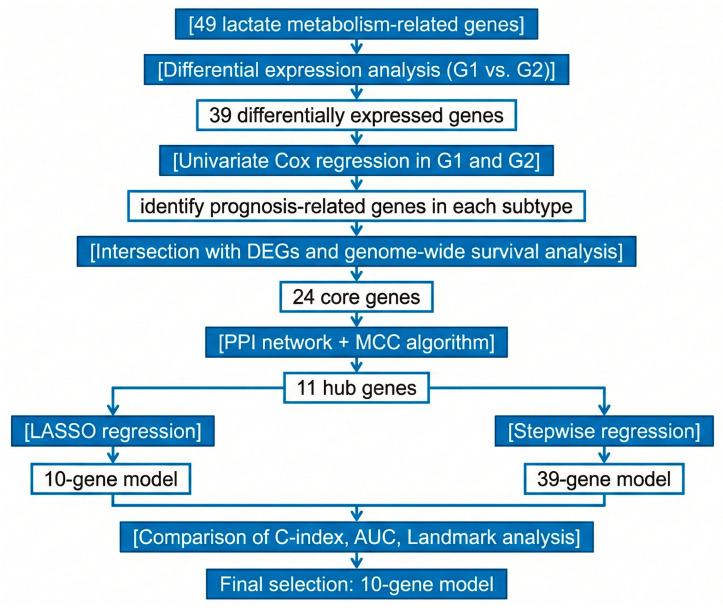
Multi-step workflow for the identification of core prognostic hub genes and the construction of the prognostic model. The flowchart details the sequential filtering process, beginning with 49 lactate metabolism-related genes (LMRGs) compiled from a three-tiered prioritization strategy. The process proceeds through subtype-specific differential expression analysis (*n* = 39 genes), prognostic screening via univariate Cox regression and genome-wide survival analysis, intersection analysis to yield 24 core candidates, and protein–protein interaction (PPI) network analysis to pinpoint the final 11 hub genes. These hub genes form the basis for LASSO regression model development and subsequent validation.

To systematically distill the most clinically relevant genes from the initial set of 49 LMRGs, we designed a multi-layer, stepwise filtering workflow. This strategy progressively refines the gene list by integrating differential expression patterns, survival associations, and protein–protein interaction (PPI) network centrality. The specific steps, also illustrated in Figure 1, were executed as follows:

Step 1: Subtype-Specific Expression Filtering.

Purpose: To identify LMRGs that are transcriptionally distinct between the newly defined G1 and G2 subtypes. We performed differential expression analysis between the G1 and G2 cohorts using the R package Limma (version 3.40.2). Multiple testing correction was performed using the Benjamini–Hochberg (BH) method to control the false discovery rate (FDR), as implemented in the topTable function. A total of 39 LMRGs exhibited significant expression differences (|log2FC| > 1 and adjusted *p*-value < 0.05) and were retained for subsequent prognostic evaluation.

Step 2: Prognostic Relevance Assessment.

Purpose: To select genes with significant associations with overall survival (OS). Univariate Cox regression analyses were conducted on the 39 differentially expressed genes within both the G1 and G2 cohorts independently. Additionally, a genome-wide univariate Cox analysis was performed to identify all prognostic genes in each subtype. This provided a broader genomic context for survival association. For all Cox regression models, the proportional hazards (PH) assumption was tested using the Schoenfeld residuals method.

Step 3: Intersection Analysis.

Purpose: To isolate a core set of genes that are simultaneously related to lactate metabolism, exhibit subtype-specific expression, and correlate with patient prognosis. By intersecting the gene lists from the previous steps (39 subtype-specific DEGs, G1/G2 univariate Cox prognostic genes, and genome-wide survival genes), we identified 24 core candidate genes. The frequency with which these genes appeared across the different selection criteria is summarized in Table 2, highlighting *SLC2A3*, *SLC16A7*, and *SLC2A2* as particularly robust candidates.

Step 4: Protein–Protein Interaction (PPI) Network Construction and Hub Gene Identification.

Purpose: To further refine the 24 core candidates and pinpoint central ‘hub’ genes within the lactate metabolism interactome. The 24 core genes were submitted to the STRING database (https://string-db.org) to construct a PPI network, with an interaction threshold set at a combined score > 0.4. The network was visualized and analyzed using Cytoscape software (version 3.9.1). To identify the most topologically significant nodes, we applied the Maximal Clique Centrality (MCC) algorithm from the cytoHubba plugin. Genes with a connectivity degree ≥ 4 were designated as hub genes. This process yielded a final set of 11 hub genes (*AKR1B1*, *HK2*, *FABP4*, *SERPINE1*, *NT5E*, *SLC2A3*, *SLC2A2*, *SLC2A1*, *NRP1*, *SLC16A7*, and *PDK4*), which served as the foundation for constructing the subsequent prognostic models.

Step 5: Multivariate Cox Regression Modeling and Comparison.

Purpose: To construct and compare prognostic models based on the identified hub genes. Based on the 11 hub genes, prognostic models were constructed using both LASSO regression (via the R package glmnet, version 4.1-8, with 10-fold cross-validation) and stepwise regression (via the step function using the Akaike information criterion, AIC) to compare their predictive performance in the training set. The final 10-gene model was selected based on optimal performance in internal validation cohorts.

### 2.6. Protein–Protein Interaction Network and Hub Gene Identification

The 24 core genes were submitted to the STRING database (https://string-db.org) to construct a PPI network, with an interaction threshold set at a combined score >0.4. The network was visualized using Cytoscape software, and the hub genes with the highest connectivity were identified using the MCC algorithm from the cytoHubba plugin, ultimately filtering down to 11 hub genes.

### 2.7. Expression Validation of Hub Genes and Clinical Relevance

Expression Difference Analysis: The Wilcoxon test was used to compare the expression differences in hub genes among the G1, G2, and normal tissue groups.

Independent Prognostic Value Assessment: A multivariate Cox regression analysis was conducted for the hub genes, and a forest plot was generated to display their hazard ratios (HR) and 95% confidence intervals (CI). Based on the results of the multivariate Cox regression, a nomogram was constructed using the R package rms (version 6.8-0) to predict the OS rates of patients at 1, 2, 3, and 4 years.

### 2.8. Construction and Comparison of Prognostic Models

Based on the 39 genes with significant expression differences mentioned above, we employed multivariate Cox regression and used the step function for bidirectional stepwise regression to select the optimal model based on the AIC criterion, thus constructing the 39-gene model.

Based on the 11 hub genes, we adopted three strategies to construct prognostic models:

1. LASSO Regression: Using the R package glmnet, version 4.1-8, we performed 10-fold cross-validation to determine the optimal penalty parameter λ by minimizing the partial likelihood deviation, and selected genes with non-zero coefficients.
2. Multivariate Cox Regression: A multivariate Cox regression analysis was conducted on the hub genes to construct a risk score model based on regression coefficients.
3. Stepwise Regression (Stepwise Cox): Building upon the multivariate Cox regression, we used the step function for bidirectional stepwise regression to select the optimal model based on the AIC criterion.

Finally, we obtained the 39-gene model and the 10-gene model. The risk score calculation formula is:

*Riskscore* = Σ (*coef__i_* × *expr__i_*).

Patients were classified into high-risk and low-risk groups based on the median risk score, and survival differences between the two groups were compared using Kaplan–Meier (KM) survival curves and the log-rank test. The time-dependent ROC curve was utilized to evaluate the model’s predictive ability over 1 to 5 years, and the AUC value was used to quantify the model’s performance.

### 2.9. Internal Validation and Comparison of Models

The performance of the 39-gene model and the 10-gene model was assessed in the G1 and G2 cohorts, respectively:

The C-index and its 95% confidence interval were calculated.

Time-dependent ROC curves were plotted, and the AUC values were calculated for 1 to 5 years.

The number of at-risk patients at each time point was counted to assess the impact of sample size on the stability of the AUC in later stages.

To further evaluate the model’s predictive ability over different time periods, landmark analyses were conducted, using 3-year and 5-year time points as landmarks to assess the model’s predictive performance (C-index and time-dependent AUC) among patients who remained alive after these landmarks.

### 2.10. Association Analysis Between the Model, Tumor Microenvironment, and Stemness

To explore the biological mechanisms underlying the risk score, we analyzed its associations with the tumor immune microenvironment and stemness index:

Immune Cell Infiltration: The abundance of immune cell infiltration for each patient was estimated using six algorithms: CIBERSORT, EPIC, TIMER, QUANTISEQ, MCP-counter, and xCell (URLs and access dates are provided in Supplementary Table S2). Spearman correlation analysis was conducted to assess the correlation between the risk score and various immune cell subpopulations, with results presented in a heatmap.

Immune Checkpoint Genes: The expression values of nine classic immune checkpoint genes (*CD274*, *CTLA4*, *LAG3*, *PDCD1*, *PDCD1LG2*, *TIGIT*, *HAVCR2*, *SIGLEC15*, *ITPRIPL1*) were extracted, and their correlation with the risk score was analyzed.

Stemness Index: The OCLR (one-class logistic regression) algorithm proposed by Malta et al. was utilized to calculate the stemness index (mRNAsi) for each sample based on mRNA expression profiles. Spearman correlation analysis was performed to explore the association between the risk score and tumor stemness.

### 2.11. External Validation

To evaluate the generalizability of the model, we conducted validation in two independent external datasets: the ACRG cohort (GSE62254, *n* = 300, platform GPL570) and the GSE26253 cohort (*n* = 432, platform GPL570).

1. Data Preprocessing: Raw expression matrices for GSE62254 and GSE26253 were downloaded from the GEO database (series matrix files). The annotation file for platform GPL570 was obtained using the R package GEOquery, version 2.70.0, and probe-level expression data were summarized to the gene level by calculating the averages.
2. Risk Score Calculation: The risk scores for each sample in both cohorts were calculated using the same 10 genes and regression coefficients as in the training set.
3. Performance Evaluation: The C-index and its standard error for the risk scores were calculated, and time-dependent ROC curves for 1, 3, and 5 years were plotted to calculate the corresponding AUC values. Log-rank tests were conducted to compare survival differences between high-risk and low-risk groups.
4. Exploratory Analysis: Given the technical platform differences between the training set (RNA-seq) and validation sets (microarray), we recalculated the risk scores after z-score standardization of gene expression data in the two cohorts to investigate the impact of platform differences on model transferability. Additionally, univariate Cox regression analyses of the 10 genes were performed in the ACRG cohort to compare the consistency of hazard ratio directions with model coefficients, and to analyze the association of risk scores with known molecular subtypes in the ACRG cohort (EMT, MSI, TP53-neg, TP53-positive).
5. Investigation of Failure Reasons: To explore potential reasons for model failure, univariate Cox regression analyses of the 10 genes were conducted to compare the consistency of hazard ratio directions with model coefficients. Furthermore, risk scores were associated with known molecular subtypes in the ACRG cohort (EMT, MSI, TP53-neg, TP53-positive) through ANOVA to assess their correlation with established biological classifications.

### 2.12. Single-Cell Transcriptomic Data Analysis

To explore the cellular sources of key prognostic genes in the GC microenvironment, this study integrated two single-cell RNA sequencing datasets for analysis.

1. Analysis of the GSE163558 Dataset: The raw expression matrix for the GSE163558 dataset was downloaded from the GEO database, which includes samples from 5 GC tissues (GSM5004180, GSM5004181, GSM5004182, GSM5004184, GSM5004185) and 1 paired normal gastric mucosa tissue (GSM5004183). Using the Seurat package (version 4.1.0), a Seurat object was constructed from the raw expression matrix, and stringent quality control (QC) was performed. The following filtering criteria were applied:

(1) Genes: Retained if detected in at least 3 cells.

(2) Cells: Retained if they met the following criteria:

① Number of detected genes (nFeature_RNA) between 200 and 6500 (to filter out empty droplets and potential doublets/multiplets).

② Total UMI count (nCount_RNA) less than 50,000 (to further mitigate doublet contamination).

③ Percentage of mitochondrial gene content (percent.mt) less than 10% (to remove low-quality or dying cells).

After applying these QC filters, a total of 11,852 high-quality single cells were retained for downstream analysis, out of an initial pool of 15,234 cells. The median number of genes detected per cell was 1547 (range: 200–6482), the median UMI count was 3850 (range: 501–48,921), and the median mitochondrial gene percentage was 5.2% (range: 0.1–9.9%).

The six samples were merged into a single Seurat object. No explicit batch correction algorithm (e.g., Harmony(version 1.0), CCA) was applied, as all samples were processed on the same 10x Genomics platform (10x Genomics, Pleasanton, CA, USA) and visual inspection of t-SNE embeddings colored by sample-of-origin showed good mixing of cells from different samples within major cell type clusters. This indicated that technical batch effects were minimal and did not drive the observed clustering.

The UMI counts were normalized using the NormalizeData function with the “LogNormalize” method and a scale factor of 10,000, and the top 2000 highly variable genes were identified using the FindVariableFeatures function (“vst” method) for principal component analysis (PCA). Based on the first 18 principal components (selected using the elbow plot heuristic), t-distributed stochastic neighbor embedding (t-SNE) was employed for dimensionality reduction and visualization. Cell clustering was achieved using the FindClusters function (resolution = 0.5).

Marker genes for each cell cluster were identified using the FindAllMarkers function with the default Wilcoxon rank-sum test. *p*-values were adjusted for multiple testing using the Bonferroni correction method based on the total number of genes tested, as implemented in Seurat’s default workflow. Each cell subgroup was classified based on the expression of canonical marker genes, with reference to annotations from the TISCH database (http://tisch.comp-genomics.org/ accessed on 10 February 2026).

2. Analysis of the GSE134520 Dataset (Gastric Tissue Verification): To further validate the expression characteristics of *FABP4* in fibroblasts, pre-processed .h5 files and corresponding cell annotation results for the GSE134520 dataset were downloaded from the TISCH database. Data processing was conducted using R software MAESTRO, version 1.6.0, and Seurat, employing the t-SNE method to re-cluster the cells, with a focus on analyzing the gene expression characteristics of each cell type in gastric tissues.

3. Data Integration and Batch Effect Assessment: The GSE163558 dataset comprises single-cell transcriptomes from 5 gastric cancer tissues and 1 paired normal gastric mucosa sample. These six samples were merged into a single Seurat object prior to normalization and downstream analysis. No explicit batch correction algorithm (e.g., Harmony (version 1.0), Seurat CCA, or MNN) was applied. This decision was based on the following rationale: (1) all samples were generated on the same 10x Genomics platform (10x Genomics, Pleasanton, CA, USA) with consistent library preparation protocols, minimizing technical variability; (2) visual inspection of t-SNE embeddings with cells colored by sample-of-origin revealed good intermixing of samples within major cell type clusters, suggesting that biological variation outweighed potential batch effects; and (3) to confirm robustness, a parallel analysis using the Harmony (version 1.0) algorithm for batch correction was performed, which produced cell clustering and marker gene expression patterns that were qualitatively identical to the uncorrected analysis, particularly with respect to the enrichment of *HK2* in NK/pDC populations and *FABP4* in proliferative CD8+ T cells and fibroblasts.

### 2.13. Association Analysis of FABP4 Mutation, Expression, and Prognosis

To further explore the potential role of *FABP4* in GC molecular subtypes, we conducted the following analyses in the G1 and G2 subtypes:

1. Tumor Mutation Burden (TMB) Correlation Analysis: TMB score for each patient in G1 and G2 was calculated, and the Spearman rank correlation test was used to assess the correlation between *FABP4* expression and TMB.
2. Mutation Characteristic Analysis: The mutation status of the *FABP4* gene was extracted from the somatic mutation data of the TCGA GC cohort, and the mutation frequency in the G2 subtype was calculated. Additionally, the expression distribution of mutated samples was analyzed in conjunction with the previously grouped expression levels of *FABP4* (high/low expression).
3. Prognostic Association Analysis: In the G2 subtype, *FABP4* expression was treated as a continuous variable, and its association with OS was evaluated using a univariate Cox proportional hazards regression model, calculating the HR and its 95% CI. All statistical analyses were performed using R software (version 4.0.3), with a two-sided *p* < 0.05 considered statistically significant.

### 2.14. Statistical Analysis

All statistical analyses were performed using R software (version 4.0.3). Survival curves were compared using the log-rank test. The Cox proportional hazards regression model was utilized to assess the association between genes or risk scores and survival time, calculating the HR and its 95% CI. Comparisons between two groups were conducted using the Wilcoxon test or Student’s *t*-test, while comparisons among multiple groups were performed using the Kruskal–Wallis test or ANOVA. A *p*-value of <0.05 was considered statistically significant.

**Figure A1:**
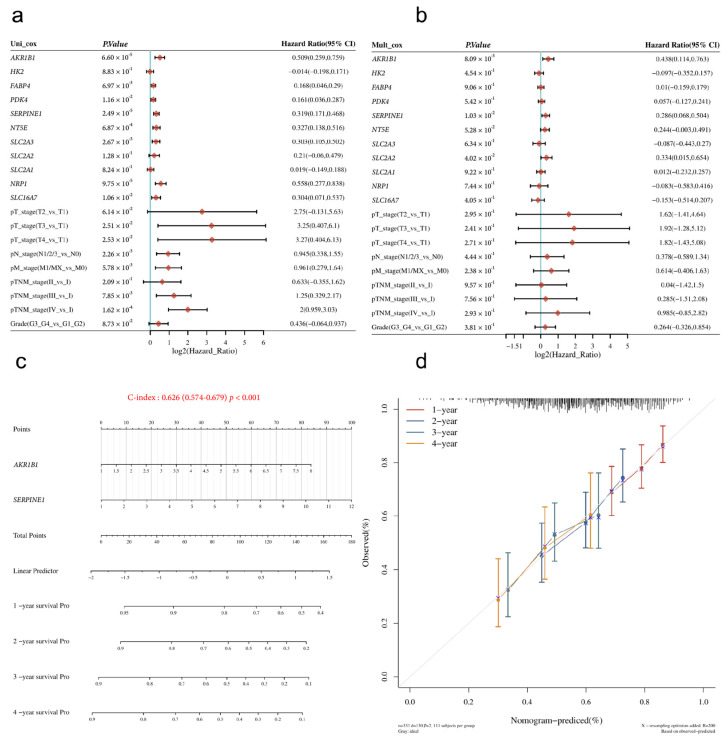
Prognostic value of hub genes: (**a**) Forest plot of univariate Cox regression analysis showing the HR and 95% CI for the 11 hub genes; (**b**) Forest plot of multivariate Cox regression analysis; (**c**) Nomogram constructed based on multivariate Cox regression to predict the OS rates of GC patients at 1 year, 2 years, and 3 years; (**d**) Calibration curves for the nomogram predicting survival rates at 1 year, 2 years, and 3 years, where the dashed diagonal line represents ideal predictions and the colored solid lines represent actual observed values.

For all constructed Cox proportional hazards regression models, Univariate and multivariate Cox regression analyses of the 11 hub genes, along with a nomogram and calibration curves, are shown in Figure A1. The proportional hazards (PH) assumption was tested using the Schoenfeld residuals method implemented in the cox.zph function of the R survival package (version 3.5-7). A non-significant global test *p*-value (*p* > 0.05) indicates that the PH assumption is not violated, supporting the validity of the model. For individual covariates showing evidence of non-proportionality, the corresponding hazard ratios should be interpreted as average effects over the follow-up period.

## 3. Results

### 3.1. Identification of Molecular Subtypes of Gastric Cancer and Clinical Pathological Characteristics

**Figure 2:**
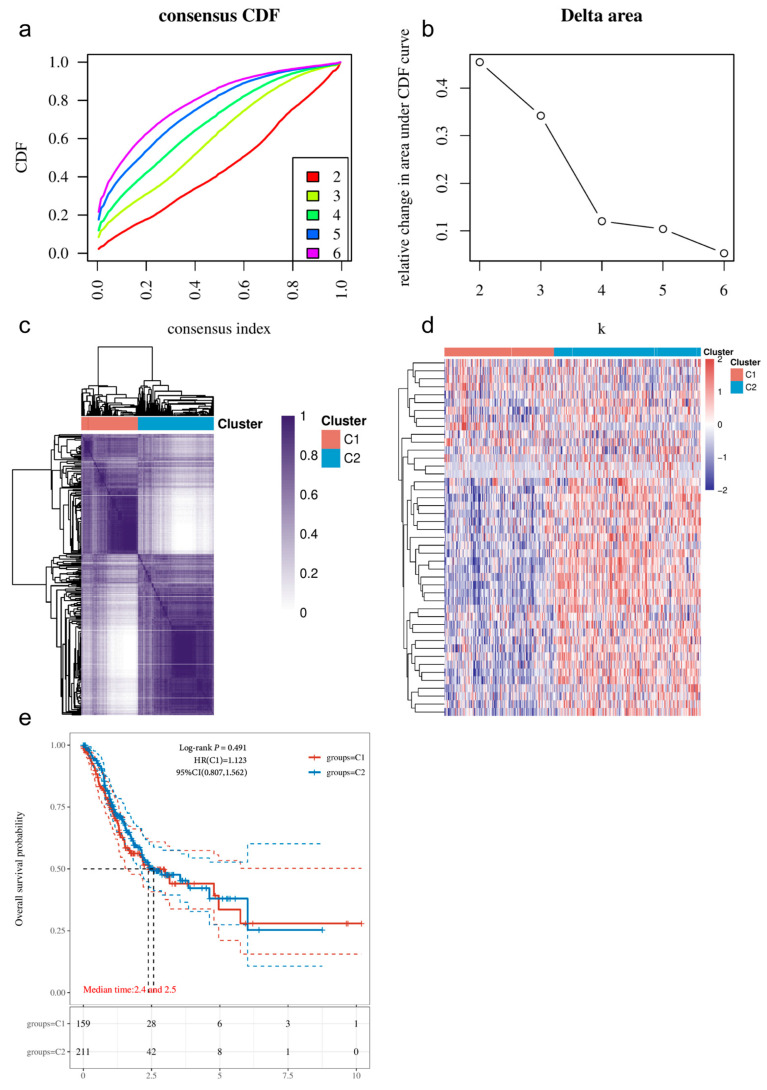
Identification of Molecular Subtypes of GC: (**a**) CDF curve; (**b**) CDF Delta area plot, indicating that k = 2 is the optimal number of clusters; (**c**) Consensus clustering matrix heatmap (k = 2), with both rows and columns representing samples, and different colors indicating different subtypes; (**d**) Expression heatmap of lactic acid metabolism-related genes in G1 and G2 subtypes (red: high expression; blue: low expression); (**e**) OS KM curve for G1 and G2 subtype patients, with *p*-values calculated using the log-rank test, showing median survival times in the figure.

Through consensus clustering analysis, we divided 375 GC samples from TCGA into two stable molecular subtypes: G1 (*n* = 160) and G2 (*n* = 215). The CDF curve and the CDF Delta area plot indicated that the area under the curve changed minimally when the number of clusters (k = 2), suggesting that this division into two subtypes is optimal (Figure 2a,b). The consensus clustering matrix heatmap clearly demonstrated the distribution of samples between the two subtypes (Figure 2c). Notably, the expression patterns of lactic acid metabolism-related genes exhibited significant differences between G1 and G2 (Figure 2d). Survival analysis revealed that patients in the G1 subtype had a shorter OS compared to those in the G2 subtype (median survival time: 2.4 years vs. 2.5 years, log-rank *p* < 0.001, Figure 2e). A comparative analysis of clinical pathological characteristics between the two subtypes is shown in Table 3, where age was found to be significantly different between the two groups (*p* = 0.026), while other indicators such as gender and TNM staging did not show significant differences (*p* > 0.05).

### 3.2. Differentially Expressed Genes and Functional Enrichment Analysis Between Subtypes

**Figure 3:**
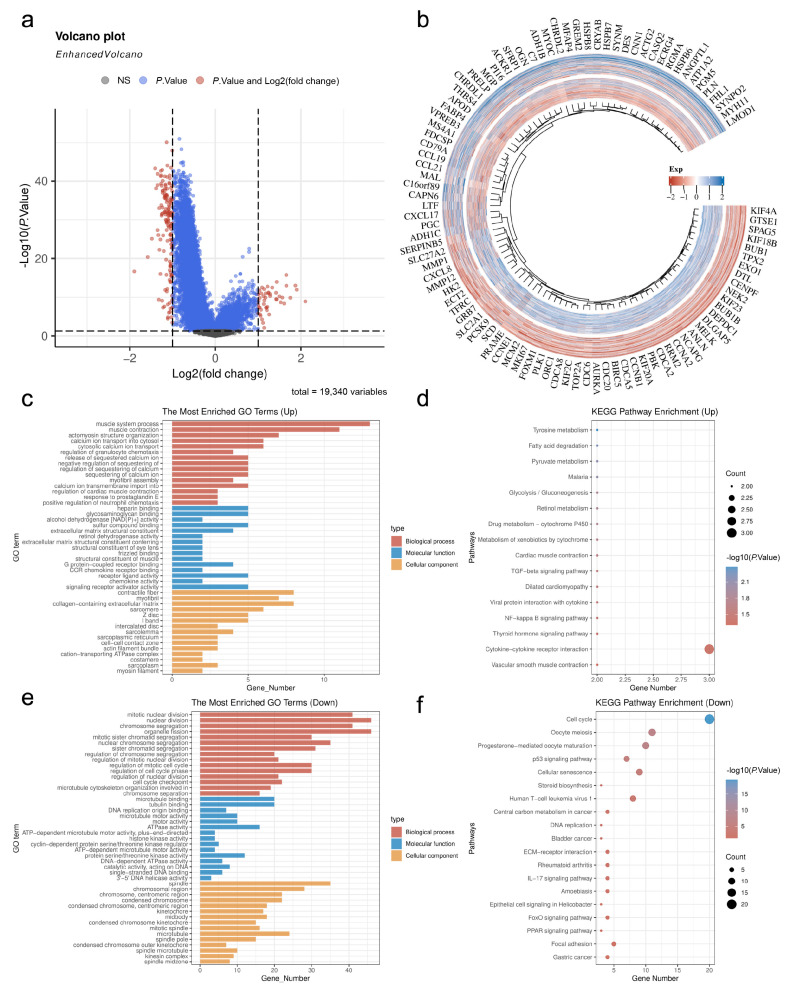
Differential Expression Analysis Between G1 and G2 Subtypes: (**a**) Volcano plot of DEGs (red: |log2FC| > 1 and adj. *p* < 0.05; blue: adj. *p* < 0.05 only; gray: no significant difference); (**b**) Expression heatmap of the top 50 most significantly differentially expressed upregulated and downregulated genes; (**c**) GO enrichment analysis of upregulated genes; (**d**) KEGG pathway enrichment analysis of upregulated genes; (**e**) GO enrichment analysis of downregulated genes; (**f**) KEGG pathway enrichment analysis of downregulated genes.

A total of 48 upregulated genes and 149 downregulated genes were identified through differential expression analysis between G1 and G2 (|log2FC| > 1, adj. *p* < 0.05). The volcano plot illustrates the overall distribution of DEGs (Figure 3a), while the heatmap displays the expression patterns of the top 50 upregulated and downregulated genes (Figure 3b). GO enrichment analysis indicated that upregulated genes were primarily enriched in biological processes related to glycolysis, extracellular matrix organization, and molecular functions such as extracellular matrix components and cell adhesion molecule binding (Figure 3c). In contrast, downregulated genes were enriched in pathways associated with immune response and lymphocyte activation (Figure 3e). KEGG pathway enrichment analysis revealed that upregulated genes were significantly enriched in glycolysis/gluconeogenesis, HIF-1 signaling pathway, and central tumor carbon metabolism (Figure 3d), whereas downregulated genes were mainly enriched in cell adhesion molecules and chemokine signaling pathways (Figure 3f). These findings suggest that the G1 subtype exhibits a microenvironment characterized by active glycolysis accompanied by immune suppression, while the G2 subtype is relatively immune-active.

### 3.3. Screening of Core Prognostic Genes and Hub Gene Identification

**Figure 4:**
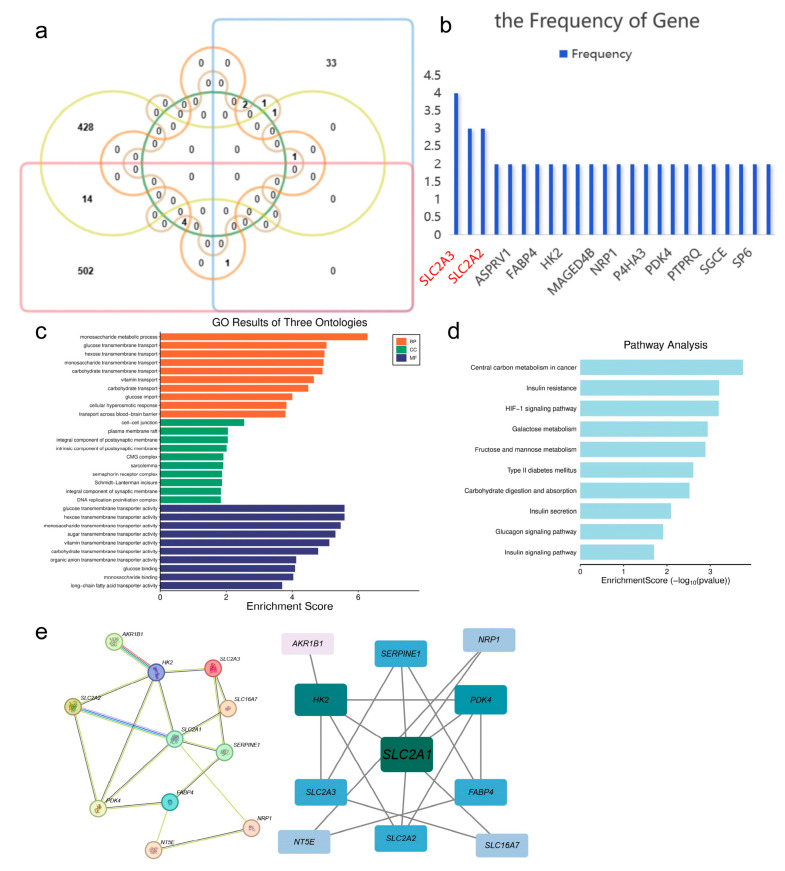
Screening of Core Genes and Functional Enrichment Analysis: (**a**) Venn diagram showing the intersection of six gene lists, resulting in a total of 24 core genes; (**b**) Bar chart displaying the frequency of the 24 core genes across each list, with genes appearing ≥ 3 times marked in red; (**c**) GO enrichment analysis of core genes (BP, CC, MF), showcasing the top 10 most significant entries; (**d**) KEGG pathway enrichment analysis of core genes; (**e**) PPI network constructed for the 24 core genes based on the STRING database, with hub genes (connectivity degree ≥ 4) shown as larger nodes.

**Figure 5:**
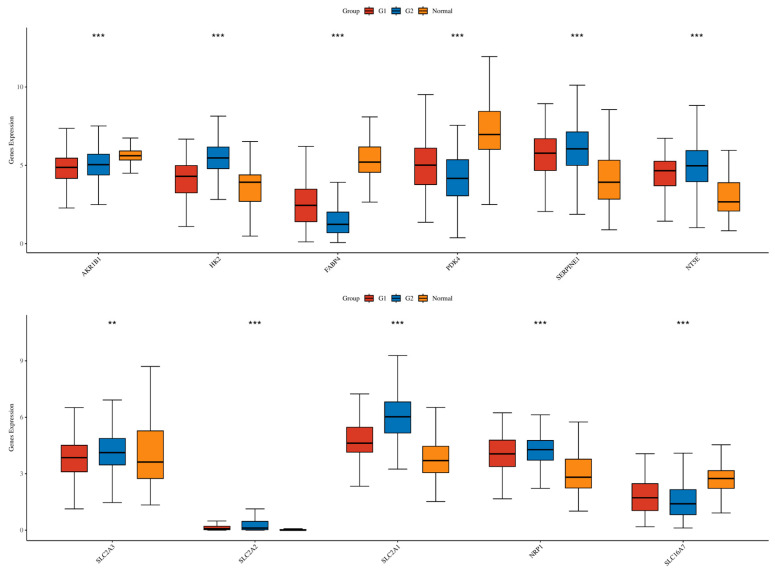
Comparison of Hub Gene Expression Between G1 and G2 Subtypes: The boxplot displays the expression levels of 11 hub genes in G1 and G2 subtypes, with *p*-values calculated using the Wilcoxon rank-sum test. ** *p* < 0.01, *** *p* < 0.001.

To identify core genes related to the prognosis of GC and involved in lactic acid metabolism, we designed a multi-layered screening process. Initially, the expression levels of 49 lactic acid metabolism-related genes were calculated, resulting in 39 genes with significantly different expression between G1 and G2 (*p* < 0.05, Figure S1). Univariate Cox regression analysis identified 3 genes (*PDK4*, *SLC2A2*, *SLC2A3*) in the G1 cohort and 2 genes (*SLC16A7*, *SLC2A3*) in the G2 cohort that were significantly associated with OS (*p* < 0.05, Figure S2). Genome-wide batch survival analysis further identified 444 prognostic-related genes in G1 and 521 in G2 (Figure S3). The intersection of the aforementioned genes with the DEGs between G1 and G2 yielded 24 core genes (Figure 4a), among which *SLC2A3*, *SLC16A7*, and *SLC2A2* appeared with higher frequency across multiple lists (Figure 4b, Table 2). GO and KEGG enrichment analysis of these 24 core genes pointed towards pathways such as glycolysis and the HIF-1 signaling pathway (Figure 4c,d). A PPI network was constructed using the STRING database, identifying 11 hub genes with a connectivity degree ≥ 4, including *AKR1B1*, *HK2*, *FABP4*, *SERPINE1*, *NT5E*, *SLC2A3*, *SLC2A2*, *SLC2A1*, *NRP1*, *SLC16A7*, and *PDK4* (Figure 4e). The boxplot demonstrated that these 11 hub genes exhibited significant differences in expression levels between G1 and G2 (Wilcoxon test, *p* < 0.05, Figure 5).

### 3.4. Single-Cell Transcriptomic Profiling Reveals the Enrichment of HK2 and FABP4 in Specific Immune/Stromal Cell Subpopulations

#### 3.4.1. Quality Control and Cell Type Annotation

To investigate the cellular origins of key LMRGs, we analyzed the GSE163558 single-cell dataset comprising gastric cancer and adjacent normal tissues. After applying stringent quality control filters (detailed in Section 2.12), a total of 11,852 high-quality single cells were retained for downstream analysis. The median number of genes detected per cell was 1547 (range: 200–6482), and the median UMI count was 3850 (range: 501–48,921). The median mitochondrial gene percentage was 5.2% (range: 0.1–9.9%), confirming the high viability of the retained cells.

**Figure 6:**
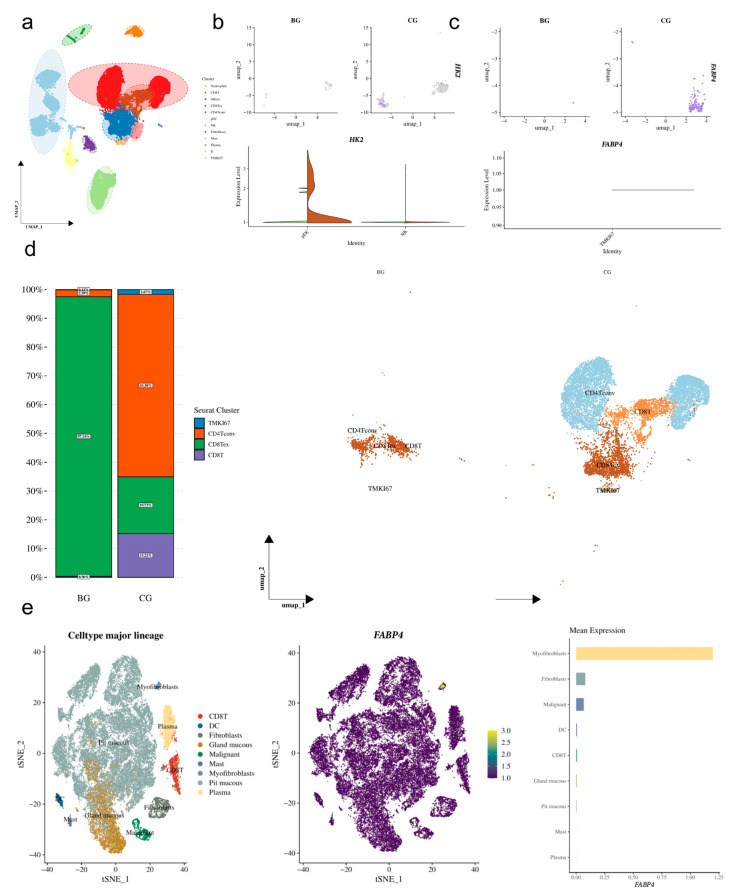
Single-Cell Transcriptomic Profiling Reveals the Enrichment of *HK2* and *FABP4* in Specific Immune/Stromal Cell Subpopulations: (**a**) t-SNE plot displaying the clustering results of all quality-controlled cells in the GSE163558 dataset, with different colors representing different cell subpopulations; (**b**) Expression feature plot of *HK2* in the t-SNE map and its expression levels in the pDC and NK cell subpopulations shown in a violin plot; (**c**) Expression feature plot of *FABP4* in the t-SNE map and its expression levels in the TMKI67 cell subpopulation displayed in a violin plot; (**d**) Co-expression analysis of *CD3D* (T cell marker) and *CD8A* (CD8+ T cell marker) in the TMKI67 subpopulation and other immune cells shown in the t-SNE plot; (**e**) t-SNE plot (**left**) and bar chart (**right**) based on the GSE134520 single-cell dataset of gastric tissue, demonstrating the specific high expression of *FABP4* in fibroblasts, validating its cellular source in gastric tissue.

Dimensionality reduction via t-SNE and graph-based clustering (resolution = 0.5) identified 10 distinct cell clusters (Figure 6a). Cell type annotation for each cluster was performed based on the expression of canonical marker genes, as detailed in Table S4. The quality control metrics and filtering thresholds are summarized in Figure S5.

#### 3.4.2. Cellular Enrichment Patterns of *HK2* and *FABP4*

Having established the cellular landscape, we next examined the expression patterns of our hub genes *HK2* and *FABP4* across these annotated clusters. Results indicated that *HK2* expression is not limited to epithelial tumor cells; rather, it is distributed across various immune cells, with a notable enrichment trend in NK cells and pDCs (Figure 6b), suggesting that *HK2* may play an important role in the metabolic activation of innate immune cells. Conversely, *FABP4* displayed a more concentrated expression pattern, primarily enriched in the *MKI67* high-expressing cell subpopulation (hereafter referred to as TMKI67), as well as in fibroblasts and macrophages (Figure 6c). Further analysis revealed that the TMKI67 subpopulation also highly expresses classic T cell markers *CD3D* and *CD8A*, as well as the effector molecule *GZMB* (Figure 6d), confirming that this subpopulation consists of actively proliferating effector CD8+ T cells. The specific high expression of *FABP4* in such cells suggests that it may support the biosynthetic and effector functions of proliferative T cells by regulating fatty acid metabolism.

Additionally, to clarify the expression pattern of *FABP4* in fibroblasts, we conducted an independent validation using the GSE134520 single-cell dataset of gastric tissue. The results showed that *FABP4* also exhibited specific high expression in fibroblasts (Figure 6e), further supporting the hypothesis that *FABP4* may participate in tumor microenvironment remodeling by influencing fibroblast function.

### 3.5. Mutations, Expression, and Prognostic Features of FABP4 in the G2 Subtype

**Figure 7:**
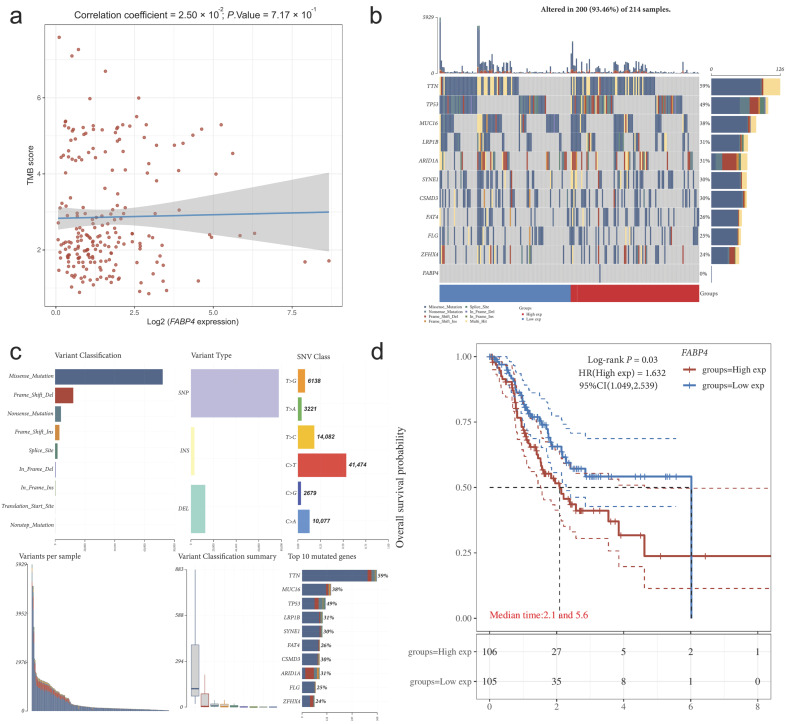
Mutations, Expression, and Prognostic Features of *FABP4* in the G2 Subtype: (**a**) Scatter plot showing the correlation between *FABP4* expression and TMB. Red markers represent *FABP4* mutated samples (TCGA-RD-A8NB-01 and TCGA-BR-8081-01). Spearman correlation analysis indicates no significant correlation (rho = −0.020, *p* = 0.770), suggesting that *FABP4* high expression is not driven by genomic instability; (**b**) Oncoplot of somatic mutation landscape in the G2 subtype. Genes are ordered by mutation frequency and samples are sorted by histological type. The top bar chart displays TMB distribution, and the right legend indicates mutation types. The positions of *FABP4* mutated samples are labeled and correspond to the high expression status; (**c**) Summary plot of mutation spectrum groups, showing the distribution of different mutation classifications, mutation types, and SNV categories, with the bottom indicating the mutation burden for each sample; (**d**) KM survival curves for the high and low *FABP4* expression groups in the G2 subtype. Univariate Cox regression analysis reveals that high expression of *FABP4* is significantly associated with poorer OS (HR = 1.71, 95% CI: 1.10–2.67, *p* = 0.017), indicating that its prognostic effect is subtype-specific.

To elucidate the potential function of *FABP4* in the G2 subtype, we first analyzed its correlation with TMB. Among the 215 patients in the G2 subtype, two cases of *FABP4* gene mutations were detected (mutation frequency 0.93%), while no mutations were found in the G1 subtype. Notably, both mutated samples belonged to the high expression group of *FABP4*, suggesting that mutation and high expression status coexist (Figure 7a). Further analysis revealed no significant correlation between *FABP4* expression and TMB in the G2 subtype (Spearman rho = −0.020, *p* = 0.770; Figure 7b,c), indicating that its high expression is not driven by genomic instability.

In terms of prognosis, univariate Cox regression analysis demonstrated that high expression of *FABP4* in the G2 subtype was significantly associated with poorer OS (HR = 1.71, 95% CI: 1.10–2.67, *p* = 0.017; Figure 7d). Importantly, this result contrasts sharply with the conclusion from a subsequent multivariate model in the overall cohort, where *FABP4* acted as a protective factor (HR < 1), suggesting that the prognostic effect of *FABP4* is subtype-specific.

### 3.6. Construction of Prognostic Model Based on Core Genes

**Figure 8:**
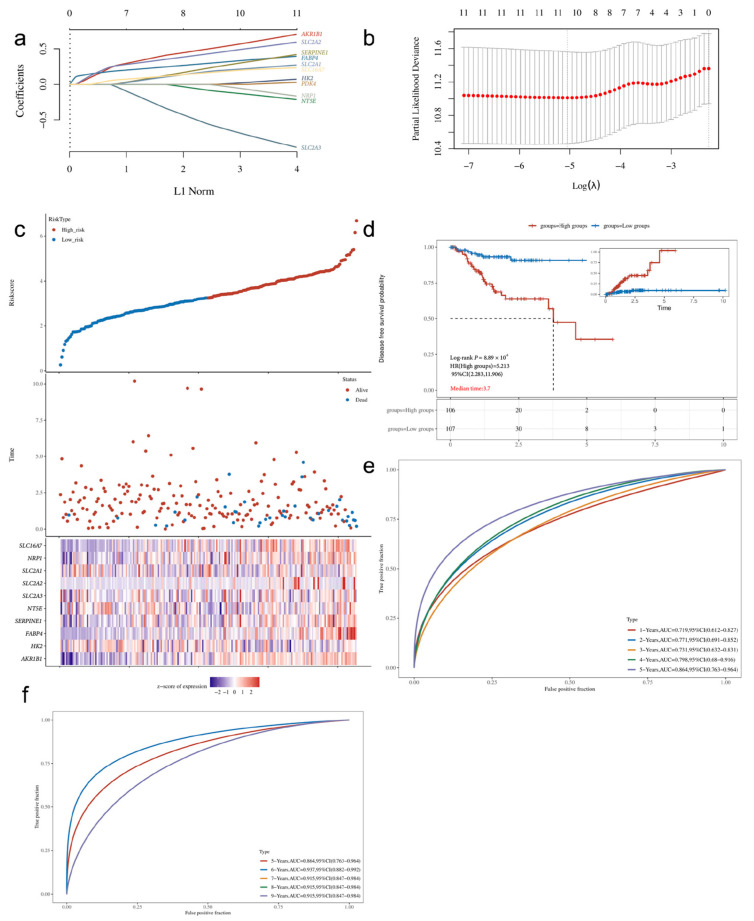
Construction and Evaluation of the 10-Gene Prognostic Model: (**a**) LASSO cross-validation error curve, with the vertical dotted line indicating the optimal λ value (λ.min = 0.0064); (**b**) Bar chart showing the coefficients of the final 10 genes (red: risk factors, blue: protective factors); (**c**) Risk score distribution of the 10-gene model in the training set, along with patient survival status and gene expression heatmap; (**d**) KM survival curves for high-risk and low-risk groups; (**e**) Time-dependent ROC curve for 1–5 year OS prediction; (**f**) Time-dependent ROC curve for 5–9 year OS prediction. Note: In subfigure (**f**), the orange and green curves overlap with the purple curve and are therefore not distinctly visible; only the purple curve is presented.

Using the aforementioned 11 hub genes, we performed LASSO regression with 10-fold cross-validation to further select variables. The optimal model, containing 10 genes, was obtained when λ = 0.0064 (Figure 8a). The coefficients from the multivariate Cox regression showed that *AKR1B1*, *SERPINE1*, *NT5E*, *SLC2A3*, *SLC2A2*, *SLC2A1*, and *NRP1* were positive coefficients (risk factors), while *HK2*, *FABP4*, and *SLC16A7* were negative coefficients (protective factors) (Figure 8b, Table 4). The risk score calculation formula is as follows:

*Riskscore* = (0.391) × *AKR1B1* + (−0.180) × *HK2* + (−0.046) × *FABP4* + (0.068) × *SERPINE1* + (0.242) × *NT5E* + (0.295) × *SLC2A3* + (0.490) × *SLC2A2* + (0.259) × *SLC2A1* + (0.001) × *NRP1* + (−0.194) × *SLC16A7* (1)

Patients were stratified into high-risk and low-risk groups based on the median risk score. KM survival curves indicated that patients in the high-risk group had significantly poorer prognoses (log-rank *p* < 0.001, Figure 8c,d). Time-dependent ROC curves for 1- to 5-year OS prediction showed AUCs ranging from 0.719 to 0.864 (Figure 8e), while the model’s performance for 5- to 9-year prediction declined due to limited sample size (Figure 8f).

### 3.7. Internal Validation Metrics

To evaluate the robustness of the 10-gene prognostic model and assess potential overfitting, we examined the internal validation metrics obtained from the LASSO cross-validation procedure. The 10-fold cross-validation yielded a cross-validated C-index of 0.698(95% CI: 0.654–0.742), which represents the model’s expected discriminative performance on new data sampled from the same population. The optimal penalty parameter was λ = 0.0064, at which point 10 of the 11 hub genes retained non-zero coefficients.

To further quantify model stability and optimism, we performed bootstrap internal validation with 1000 resamples. The apparent C-index (model performance on the training data) was 0.738, while the optimism-corrected C-index was 0.721, with an estimated optimism of 0.017. The close agreement between the cross-validated C-index (0.698), the optimism-corrected C-index (0.721), and the apparent C-index (0.738) indicates that the model’s performance is stable and that overfitting is minimal. Detailed bootstrap validation results are provided in Table S6.

### 3.8. Internal Validation and Proportional Hazards Assumption Testing

To ensure the robustness of our Cox regression models, we tested the proportional hazards (PH) assumption. For the final 10-gene multivariate Cox model, the global Schoenfeld residual test was not significant (*p* = 0.13), indicating that the PH assumption holds for the model as a whole. Test results for individual genes are provided in Table S3. Of note, the PH assumption was borderline for *FABP4* (*p* = 0.042), suggesting that its hazard ratio should be interpreted as an average effect over the follow-up period. This does not invalidate the model but warrants caution in interpreting the precise magnitude of the *FABP4* coefficient over extended timeframes.

**Figure 9:**
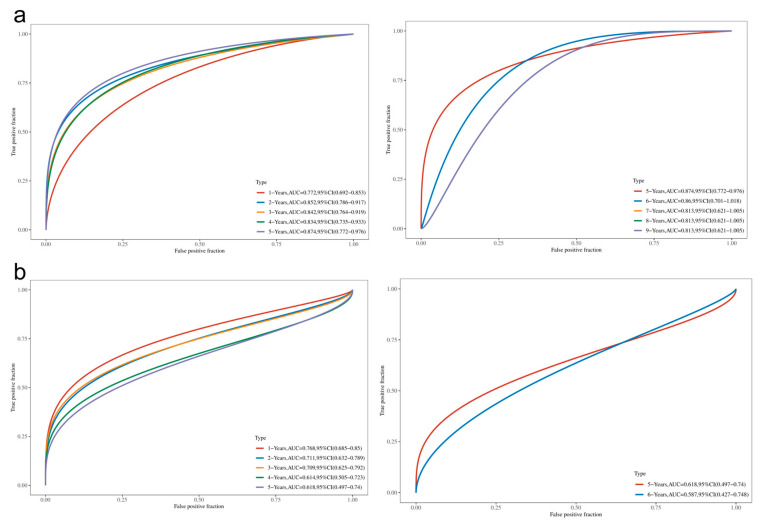
Internal Validation of the 10-Gene Model: Time-dependent ROC curves of the 10-gene model in the G1 (**a**) and G2 (**b**) validation cohorts for OS prediction from 1 to 9 years, with AUC values labeled at each time point. Note: The 5-year and subsequent AUC values for the G2 cohort are based on a severely limited number of at-risk patients (≤6 cases, as detailed in Table S1) and are therefore statistically unstable. These values are shown for completeness but should not be overinterpreted. For subfigure (**a**), the orange and green curves overlap with the purple curve and are therefore not distinctly visible; only the purple curve is presented.

In the internal validation cohort, the C-index for the 10-gene model in the G1 cohort was 0.712 (95% CI: 0.585–0.839), with 1, 3, and 5-year AUCs of 0.772, 0.842, and 0.874, respectively. In the G2 cohort, the C-index was 0.738 (95% CI: 0.599–0.877), with 1, 3, and 5-year AUCs of 0.768, 0.709, and 0.618, respectively (Figure 9, Table 5). It is important to note that the 5-year AUC for the G2 cohort is based on a very small number of patients at risk (n ≤ 6), which renders the estimate statistically unstable and should be interpreted with extreme caution. The distribution of risk scores, patient survival status, and gene expression heatmaps for the gene model in G1 and G2 can be found in Figure 4. Due to the small number of high-risk patients in the G2 cohort after five years (≤6 cases), the 5-year AUC should be interpreted with caution. Detailed time-dependent AUCs and risk counts for both models in G1 and G2 are presented in Table S1.

To ensure the robustness of our Cox regression models, we tested the proportional hazards (PH) assumption using the Schoenfeld residuals method. For the final 10-gene multivariate Cox model, the global Schoenfeld test was not significant (*p* = 0.13), indicating that the PH assumption holds for the model as a whole. Test results for individual genes are provided in Table S3. The distribution of risk scores, patient survival status, and gene expression heatmaps for the 10-gene model in the G1 and G2 cohorts is provided in Figure S4.

### 3.9. Challenges in External Validation of the Model and Heterogeneous Effects of Key Genes

**Figure 10:**
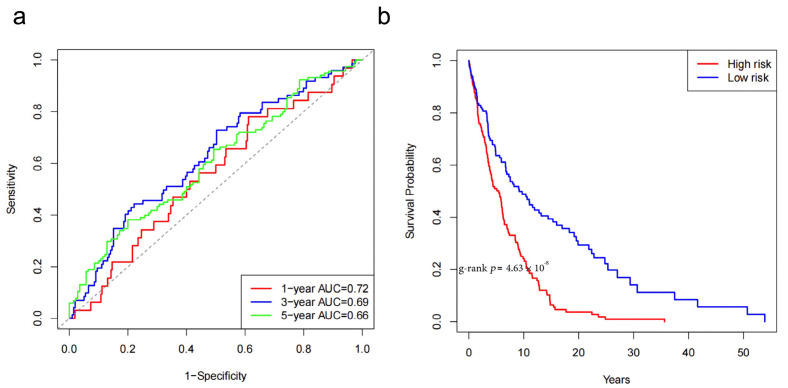
Predictive Performance of the 10-Gene Prognostic Model in the GSE26253 External Validation Cohort: (**a**) Time-dependent ROC curves assessing the model’s performance in predicting OS at 1, 3, and 5 years in the GSE26253 cohort; (**b**) KM survival curves categorizing patients in the GSE26253 cohort into high-risk and low-risk groups based on the median risk score of the 10-gene model.

To evaluate the generalizability of the model, we validated the 10-gene model in two independent external microarray datasets (ACRG and GSE26253). The results showed a significant loss of predictive capability in the ACRG cohort (GSE62254) (C-index = 0.503, 95% CI: 0.454–0.552), with time-dependent AUCs of 0.568, 0.489, and 0.481 for 1, 3, and 5 years, respectively (Table 6). In contrast, the model demonstrated some predictive ability in the GSE26253 cohort (C-index = 0.603, 95% CI: 0.562–0.644), but it still fell below clinically acceptable levels (Figure 10).

To investigate the reasons for the model’s failure, we performed univariate Cox regression analysis of the 10 genes in the ACRG cohort (Table 7). We found that the risk ratios for three genes—*AKR1B1*, *FABP4*, and *SLC16A7*—were in the opposite direction compared to the coefficients in the model. Notably, *FABP4* acted as a strong risk factor in the ACRG cohort (HR = 2.64, 95% CI: 1.51–4.61, *p* = 0.001), while it was a protective factor in the multivariate model of the overall TCGA cohort (HR = 0.955). Importantly, this risk effect direction is highly consistent with the *FABP4* risk effect we observed internally in the G2 subtype (HR = 1.71) (Figure 7b), further confirming the subtype specificity of this gene’s function. This finding suggests that prognostic models constructed based on bulk transcriptomics may fail due to the reverse effects of key genes across different subtypes or populations, and it also reveals the complexity of the functional roles of LMRGs.

### 3.10. Association Analysis of Risk Scores with Tumor Microenvironment and Stemness

**Figure 11:**
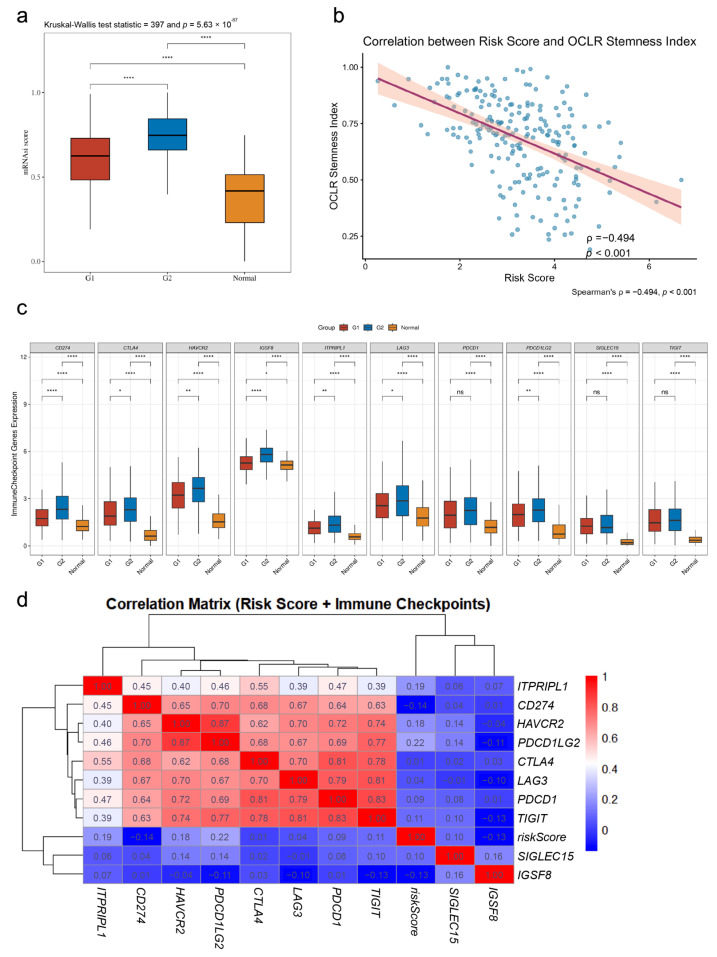
Association of the 10-Gene Model with Stemness Index and Immune Checkpoints: (**a**) Comparison of the OCLR stemness index (mRNAsi) between G1, G2 subtypes, and normal tissues; (**b**) Scatter plot showing the correlation between risk scores and mRNAsi, with Spearman correlation coefficients and *p*-values indicated; (**c**) Box plots of immune checkpoint gene expression (*PDCD1*, *CD274*, *CTLA4*, etc.) in G1, G2, and normal tissues; (**d**) Heatmap of the correlation between risk scores and immune checkpoint gene expression. Significance levels for panel (**c**): * *p* < 0.05, ** *p* < 0.01, **** *p* < 0.0001.

**Figure 12:**
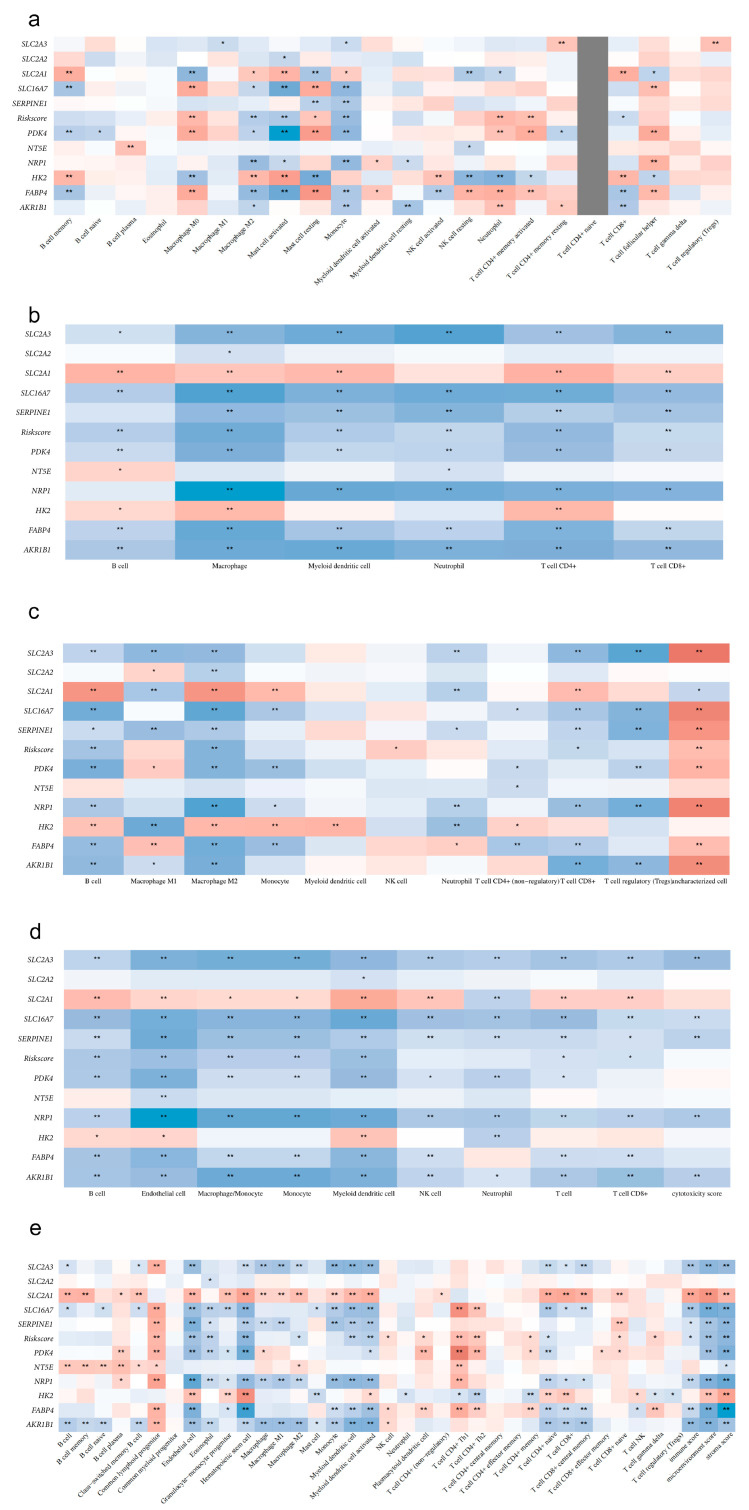
Multi-Algorithm Validation of the 10-Gene Model with Immune Cell Infiltration. The heatmap shows the Spearman correlation between risk scores and immune cell infiltration scores estimated by five different algorithms: (**a**) EPIC; (**b**) TIMER; (**c**) QUANTISEQ; (**d**) MCP-counter; (**e**) xCEL. Colors represent Spearman correlation coefficients (red: positive correlation; blue: negative correlation; intensity indicates magnitude). * *p* < 0.05, ** *p* < 0.01.

To further explore the biological significance behind the risk scores, we analyzed their associations with the tumor immune microenvironment and stemness index. The stemness index analysis revealed a significant difference in OCLR mRNAsi between G1 and G2 subtypes (Figure 11a), and the risk score showed a positive correlation with mRNAsi (Spearman r = 0.42, *p* < 0.001, Figure 11b). The expression of immune checkpoint genes in G1, G2, and normal tissues is shown in Figure 11c, with the risk score significantly associated with the expression of multiple immune checkpoint genes, including *PDCD1*, *CD274*, and *CTLA4* (Figure 11d). Multi-algorithm immune infiltration analysis indicated a significant correlation between risk scores and the abundance of various immune cells, such as M0 macrophages, cancer-associated fibroblasts (CAFs), and myeloid cells (Figure 12, Table A1), suggesting that the model may be related to the remodeling of the tumor immune microenvironment.

### 3.11. Stability and Sensitivity Analyses of the Prognostic Model

To further investigate the robustness of the 10-gene model and to dissect potential causes underlying its failure in external validation, we performed two supplementary analyses.

#### 3.11.1. Repeated Cross-Validation Stability

To assess the stability of the model’s performance within the TCGA training cohort, we conducted 100 iterations of 5-fold cross-validation. Across the 500 validation folds, the median C-index was 0.71 (interquartile range [IQR]: 0.67–0.75; range: 0.59–0.82) (Table S7). The distribution is centered near the apparent C-index (0.738) and the cross-validated C-index (0.698), indicating that the model’s discriminative ability is reasonably robust to random data partitioning. However, the observed variability (IQR width of 0.08) suggests that performance estimates are sensitive to sample composition, reflecting moderate cohort dependency.

#### 3.11.2. Sensitivity Analysis Excluding *FABP4*

Given the prominent role of *FABP4* in the observed subtype-specific effect reversal, we tested whether excluding *FABP4* from the model could improve performance in the ACRG cohort. The entire LASSO Cox regression pipeline was repeated after removing *FABP4* from the initial pool of 11 hub genes. In the ACRG cohort, the resulting 9-gene model yielded a C-index of 0.51 (95% CI: 0.46–0.56) and time-dependent AUCs of 0.57, 0.49, and 0.48 at 1, 3, and 5 years, respectively (Table S8). These values are nearly identical to those of the original 10-gene model (C-index = 0.503), indicating that the exclusion of *FABP4* does not meaningfully rescue model performance. This finding suggests that the failure of external validation is not attributable to a single gene’s effect reversal but rather reflects broader incompatibilities between the TCGA-derived coefficients and the ACRG data structure.

## 4. Discussion

This study categorized GC into two molecular subtypes (G1 and G2) with significant prognostic differences based on LMRGs through consensus clustering. It systematically characterized the transcriptomic features, functional enrichment pathways, and immune microenvironment differences between the subtypes. Further multi-level gene screening and single-cell transcriptome analysis revealed that the key gene *FABP4* was specifically enriched in proliferative CD8+ T cells and fibroblasts, exhibiting a discordant prognostic effect within the G2 subtype compared to the overall model, indicating the complexity and subtype specificity of lactate metabolism-related gene functions. This research provides new data resources for understanding the metabolic heterogeneity of GC and offers a theoretical basis for the development of future precision classification strategies [13].

Through consensus clustering, we divided GC into G1 and G2 subtypes based on the expression of 49 LMRGs, which exhibited distinct expression patterns, with subtype G1 showing significantly worse prognosis than G2. Functional enrichment analysis demonstrated that upregulated genes in subtype G1 were enriched in glycolysis, HIF-1 signaling pathways, and central carbon metabolism in tumors, while downregulated genes were enriched in immune response and lymphocyte activation pathways. This result suggests that subtype G1 may present a “glycolytically active accompanied by immune suppression” microenvironment phenotype, whereas subtype G2 is relatively immune-activated.

Previous studies have confirmed that lactate metabolism reprogramming can drive immune evasion through various mechanisms: tumor-derived lactate can promote the polarization of tumor-associated macrophages to the M2 type [14], inhibit the effector functions of T cells and NK cells [15], and act as a signaling molecule regulating gene expression through histone lactylation modification. Therefore [16], the G1/G2 classification not only reflects differences in lactate metabolism states but may also represent two distinct tumor microenvironment states, providing potential stratification criteria for optimizing future immunotherapeutic strategies [17,18,19,20].

In this study, *FABP4* exhibited a complex and even contradictory prognostic effect, which is the most noteworthy finding worth exploring in depth. In the multivariable model constructed based on the overall cohort, *FABP4* acted as a protective factor (HR < 1). However, within the G2 subtype, high expression of *FABP4* was significantly associated with poor prognosis (HR = 1.71). More importantly, in the external validation cohort ACRG, the direction of the risk ratio for *FABP4* also reversed, acting as a strong risk factor (HR = 2.64), and this effect was highly consistent with our observations within the G2 subtype.

Single-cell transcriptomic data provide a possible explanation for this contradictory phenomenon. We found that *FABP4* is not unique to a single cell type but is also highly expressed in two functionally distinct cell populations: proliferative CD8+ T cells (TMKI67+) and fibroblasts. Based on this, we propose the following hypothesis: the net effect of *FABP4* may depend on which cell type predominates. In proliferative CD8+ T cells, *FABP4* may enhance anti-tumor immunity by regulating fatty acid metabolism to support T cell biosynthesis and effector functions, thus exhibiting a protective effect. In contrast, in fibroblasts, high expression of *FABP4* may promote the activation of CAFs, remodel the extracellular matrix, and subsequently facilitate tumor invasion and progression, thereby exhibiting a risk effect [21]. The balance between these two effects may shift due to differences in tumor subtypes, microenvironmental conditions, or population backgrounds.

In the G2 subtype, the risk effect of fibroblast-derived *FABP4* may have become dominant, leading to its overall pro-cancerous role. This speculation aligns with the direction of the risk effect of *FABP4* in the ACRG cohort and explains why *FABP4* is averaged out as a protective factor in the overall cohort (a mix of G1 and G2)—likely because the protective effect from T cells in the G1 subtype is more pronounced. This finding suggests that caution is required when interpreting prognostic models constructed from bulk transcriptomic data, as the overall effect of a gene may obscure its opposing roles in specific cell types. The subtype-specific effects of *FABP4* revealed in this study provide an important warning for future precise targeting of this molecule at the single-cell level [22].

In addition to *FABP4*, the 10-gene model in this study also includes several genes closely related to lactate metabolism. Members of the SLC2A family (*SLC2A1*/GLUT1, *SLC2A2*/GLUT2, *SLC2A3*/GLUT3), known as glucose transporters, are all risk factors in the model, consistent with previous studies reporting their roles in promoting proliferation and being associated with poor prognosis in various tumors [23]. *HK2*, a rate-limiting enzyme in glycolysis, is traditionally considered an oncogene; however, it acts as a protective factor in this model. Single-cell data show that *HK2* is enriched in NK cells and pDCs, suggesting that its protective effect may stem from metabolic activation of immune cells rather than the metabolic state of cancer cells themselves. Similarly, *SLC16A7* (MCT2), as a lactate transporter, also acts as a protective factor in the model, but its risk direction reverses in the ACRG cohort, indicating that its function may also depend on cell type. These findings further emphasize the importance of distinguishing between “intrinsic tumor cell” and “microenvironment cell intrinsic” gene functions in cancer research.

Another significant finding of this study is that the 10-gene prognostic model constructed based on TCGA RNA-seq data performed poorly in cross-platform external validation: it was completely ineffective in the ACRG cohort (C-index ≈ 0.5) and showed low predictive accuracy in the GSE26253 cohort (C-index = 0.603). This is not an isolated instance from our study but rather a common challenge faced in the field of multi-gene prognostic biomarkers [24,25,26]. Through a thorough analysis of the reasons for this failure, we identified two main sources of the problem:

Firstly, the differences in technical platforms [27]. TCGA data are based on RNA-seq platforms, while ACRG and GSE26253 are based on Affymetrix GPL570 chip platforms [28]. There are systematic biases in probe design, dynamic range, background noise, and other aspects across different platforms. Even after normalization, it is challenging to completely eliminate the impact on gene expression measurements.

Secondly, a more fundamental biological reason—the prognostic effects of key genes may reverse across different subtypes or populations [29,30]. In this study, the risk directions of three genes (*AKR1B1*, *FABP4*, *SLC16A7*) in the ACRG cohort were opposite to the model coefficients, directly leading to the failure of the composite score [31]. The effect reversal of *FABP4* is particularly typical and is highly consistent with our observations within the G2 subtype, suggesting that this reversal has a biological basis rather than being merely technical noise [32]. This finding warns us that prognostic models constructed from a single dataset, even if they perform well in internal validation, may fail to generalize due to ignoring tumor heterogeneity.

These observations further emphasize that future prognostic model construction must take into account the molecular heterogeneity of tumors and should undergo rigorous validation in large cohorts across multiple centers and platforms. A potential direction for improvement is to develop subtype-specific prognostic models to avoid the effect cancellation that can lead to model failure.

The most intriguing and paradoxical finding of this study is the contextual reversal of *FABP4*’s prognostic effect. Within the overall TCGA cohort and the constructed multivariate model, *FABP4* emerged as a protective factor (HR < 1). However, this effect was inverted within the G2 subtype, where high *FABP4* expression was a significant risk factor (HR = 1.71, *p* = 0.017). Strikingly, this risk association was replicated and amplified in the independent ACRG external validation cohort (HR = 2.64, *p* = 0.001). This consistency across two independent datasets in revealing a risk effect for *FABP4*—a gene traditionally considered protective in our own model—demands a deeper biological explanation.

Our integrated single-cell transcriptomic analysis offers a plausible resolution to this apparent contradiction. We observed that *FABP4* expression is not confined to a single cellular compartment but is instead enriched in two functionally antagonistic cell populations: proliferative CD8+ T cells (TMKI67+) and fibroblasts (Figure 6). This dual cellular origin provides the foundation for our proposed “balance of effects” hypothesis, wherein the net prognostic impact of *FABP4* is dictated by the relative dominance of these two cell types within a given tumor microenvironment.

On one hand, *FABP4* expression in proliferative CD8+ T cells may support anti-tumor immunity. Based on prior literature, fatty acid-binding proteins are thought to be critical for the metabolic fitness of effector T cells, where they could facilitate the uptake and intracellular trafficking of long-chain fatty acids essential for biosynthesis during clonal expansion [33,34]. We hypothesize that in a context where these proliferating T cells are abundant, *FABP4* expression might serve as a surrogate marker of a robust anti-tumor response, thereby conferring a protective effect.

On the other hand, *FABP4* expression in fibroblasts could potentially promote tumor progression. *FABP4* is a known marker of adipocytes, but its expression in cancer-associated fibroblasts (CAFs) has been documented [35]. In CAFs, it has been speculated that *FABP4* may contribute to a pro-tumorigenic microenvironment by modulating lipid metabolism, which might influence the secretion of inflammatory cytokines and extracellular matrix components [36]. Therefore, we speculate that in a microenvironment where CAFs are the predominant source of *FABP4*, its high expression could be associated with a desmoplastic, tumor-permissive stroma, thus acting as a risk factor.

To provide additional support for this hypothesis within our data, we performed a correlative analysis within the G2 subtype. We found that *FABP4* expression exhibited a significantly stronger positive correlation with a canonical CAF gene signature (Spearman’s ρ = 0.48, *p* < 0.001) than with a proliferative CD8+ T cell signature (ρ = 0.19, *p* = 0.005) (Figure S5, Table S5). This suggests that in the G2 subtype, the transcriptional signal of *FABP4* is more heavily weighted by the stromal/fibroblast compartment, potentially explaining why its net effect tips toward being a risk factor in this specific context. This subtype-specific functional reversal of *FABP4* underscores the perils of interpreting bulk transcriptomic biomarkers without a cellular-resolution lens and highlights the necessity of developing single-cell-informed prognostic tools for precision oncology.

This study has the following limitations: First, all analyses were based on public databases, lacking independent prospective cohort validation. Second, the sources of single-cell data are limited, and the functional inferences of *FABP4* in specific cell subpopulations still require further experimental validation [37]. Third, due to the inability to conduct wet lab experiments, our interpretations of gene functional heterogeneity remain at the level of inference. Finally, a critical limitation of this study is the absence of direct functional validation. While our single-cell transcriptomic data reveal the co-localization of *FABP4* in proliferative CD8+ T cells and fibroblasts, and our bulk analyses demonstrate a robust subtype-specific reversal of its prognostic association, these findings are entirely correlative. The mechanistic hypothesis we propose—that the net effect of *FABP4* depends on the relative abundance of its immune versus stromal cellular sources—remains speculative and requires rigorous experimental validation. Future studies employing cell-type-specific CRISPR knockouts, blocking antibodies, or metabolic flux assays in co-culture systems are essential to establish causality and confirm the functional roles of *FABP4* in these distinct cellular compartments.

This study successfully categorized GC into two molecular subtypes (G1/G2) with different metabolic-immune characteristics based on LMRGs and systematically characterized the transcriptomic differences and microenvironment features between these subtypes [38]. Through in-depth investigation of key genes, we found that *FABP4* is specifically expressed in proliferative CD8+ T cells and fibroblasts, revealing its opposing prognostic effects within the G2 subtype and across different datasets. This highlights the complexity and subtype specificity of lactate metabolism gene functions [39]. This research provides new insights for the precise classification of GC and offers an important reference based on heterogeneity for future targeted therapeutic strategies aimed at lactate metabolism.

## Figures and Tables

**Table 1:** Gene Set Related to Lactate Metabolism.

Gene Classification	Gene Symbol	Full Gene Name (from NCBI)
Lactate Dehydrogenase	*LDHA*	Lactate Dehydrogenase A
	*LDHB*	Lactate Dehydrogenase B
	*LDHC*	Lactate Dehydrogenase C
	*LDHD*	Lactate Dehydrogenase D
Monocarboxylate Transporter	*SLC16A1(MCT1)*	Solute Carrier Family 16 Member 1
	*SLC16A3(MCT4)*	Solute Carrier Family 16 Member 3
	*SLC16A4(MCT5)*	Solute Carrier Family 16 Member 4
	*SLC16A7(MCT2)*	Solute Carrier Family 16 Member 7
	*SLC16A8(MCT3)*	Solute Carrier Family 16 Member 8
Pyruvate Dehydrogenase Kinase	*PDK1*	Pyruvate Dehydrogenase Kinase 1
	*PDK2*	Pyruvate Dehydrogenase Kinase 2
	*PDK3*	Pyruvate Dehydrogenase Kinase 3
	*PDK4*	Pyruvate Dehydrogenase Kinase 4
Pyruvate Dehydrogenase Complex	*PDHA1*	Pyruvate Dehydrogenase E1 Subunit Alpha 1
	*PDHB*	Pyruvate Dehydrogenase E1 Subunit Beta
	*DLAT*	Dihydrolipoamide S-Acetyltransferase
	*DLD*	Dihydrolipoamide Dehydrogenase
	*PDP1*	Pyruvate Dehydrogenase Phosphatase 1
	*PDP2*	Pyruvate Dehydrogenase Phosphatase 2
Pyruvate Carboxylase	*PC*	Pyruvate Carboxylase
Pyruvate Kinase	*PKM*	Pyruvate Kinase M1/M2
	*PKLR*	Pyruvate Kinase L/R
Hexokinase	*HK1*	Hexokinase 1
	*HK2*	Hexokinase 2
	*HK3*	Hexokinase 3
Glucose Transporter	*SLC2A1(GLUT1)*	Solute Carrier Family 2 Member 1
	*SLC2A2(GLUT2)*	Solute Carrier Family 2 Member 2
	*SLC2A3(GLUT3)*	Solute Carrier Family 2 Member 3
	*SLC2A4(GLUT4)*	Solute Carrier Family 2 Member 4
Key Enzymes of Glycolysis	*GAPDH*	Glyceraldehyde-3-Phosphate Dehydrogenase
	*PGK1*	Phosphoglycerate Kinase 1
	*PGAM1*	Phosphoglycerate Mutase 1
	*ENO1*	Enolase 1
	*ENO2*	Enolase 2
	*ENO3*	Enolase 3
	*ALDOA*	Aldolase A
	*ALDOB*	Aldolase B
	*ALDOC*	Aldolase C
	*TPI1*	Triosephosphate Isomerase 1
	*PFKP*	Phosphofructokinase, Platelet
	*PFKL*	Phosphofructokinase, Liver
	*PFKM*	Phosphofructokinase, Muscle
Other Related Genes	*G6PD*	Glucose-6-Phosphate Dehydrogenase
	*MCTP1*	Multiple C2 And Transmembrane Domain Protein 1
	*MCTP2*	Multiple C2 And Transmembrane Domain Protein 2

**Table 2:** Frequency of Occurrence of 24 Core Genes in Six Gene Lists.

Gene	Frequency	Affiliated List
*SLC2A3*	4	Lactic, Bulk1, Cox2, Cox1
*SLC16A7*	3	Lactic, Bulk2, Cox2
*SLC2A2*	3	Lactic, Bulk1, Cox1
*AKR1B1*	2	Bulk2, Bulk1
*ASPRV1*	2	Bulk2, Bulk1
*CDH6*	2	Bulk2, Bulk1
*FABP4*	2	Diff, Bulk2
*GPA33*	2	Diff, Bulk2
*HK2*	2	Diff, Lactic
*KCND2*	2	Bulk2, Bulk1
*MAGED4B*	2	Bulk2, Bulk1
*MCM2*	2	Diff, Bulk2
*NRP1*	2	Bulk2, Bulk1
*NT5E*	2	Bulk2, Bulk1
*P4HA3*	2	Bulk2, Bulk1
*PCDHB5*	2	Bulk2, Bulk1
*PDK4*	2	Lactic, Cox1
*PPP1R3B*	2	Bulk2, Bulk1
*PTPRQ*	2	Bulk2, Bulk1
*SERPINE1*	2	Bulk2, Bulk1
*SGCE*	2	Bulk2, Bulk1
*SLC2A1*	2	Diff, Lactic
*SP6*	2	Diff, Bulk2
*TMSB15A*	2	Bulk2, Bulk1

Note: Diff: DEGs between G1 and G2 subtypes; Lactic: Lactic acid metabolism-related gene set; Bulk2: Significant genes from G2 cohort genome-wide batch survival analysis; Bulk1: Significant genes from G1 cohort genome-wide batch survival analysis; Cox2: Prognostic-related genes from univariate Cox regression analysis in the G2 group; Cox1: Prognostic-related genes from univariate Cox regression analysis in the G1 group. The frequency indicates the number of times a gene appears in the five lists mentioned above, excluding the lactic acid metabolism gene set. Based on the summary table data, *SLC2A3*, *SLC16A7*, *SLC2A2*, and *PDK4* appear in the univariate Cox lists, while the other genes with a frequency of 2 are present in both genome-wide batch survival analysis lists (Bulk1 and Bulk2). Some genes also appear in the differentially expressed gene list or the lactic acid metabolism gene set, with specific distributions as indicated above.

**Table 3:** Comparison of Clinical Pathological Characteristics Between G1 and G2 Subtype Patients.

	Charar	C1	C2	*p*-Value
Status	Alive	96	132	
	Dead	64	83	0.868
Age	Mean (SD)	64.4 (10.7)	66.9 (10.5)	
	Median [MIN, MAX]	65 [41, 90]	68 [35, 90]	0.026
Gender	FEMALE	54	80	
	MALE	106	135	0.56
Race	ASIAN	41	33	
	BLACK	2	9	
	WHITE	103	135	
	ISLANDER		1	0.035
pT_stage	T1a	1	1	
	T1b	4	8	
	T2	24	34	
	T2a	4	5	
	T2b	4	9	
	T3	80	88	
	T4	11	19	
	T4a	18	28	
	T4b	12	12	
	TX	2	6	
	T1		5	0.824
pN_stage	N0	41	70	
	N1	46	51	
	N2	30	45	
	N3	11	15	
	N3a	22	20	
	N3b	4	2	
	NX	5	11	0.37
pM_stage	M0	139	191	
	M1	12	13	
	MX	9	11	0.829
pTNM_stage	IA	4	10	
	IB	12	25	
	II	11	16	
	IIA	19	16	
	IIB	21	28	
	IIIA	23	37	
	IIIB	28	24	
	IIIC	17	18	
	IV	14	24	
	I		2	
	III		3	0.358
Grade	G1	5	5	
	G2	47	90	
	G3	103	116	
	GX	5	4	0.092
New tumor event type	Metastasis	26	28	
	Metastasis:Recurrence	1	3	
	Primary	1	2	
	Recurrence	11	18	
	Recurrence:Primary		1	0.677
Radiation therapy	Non-radiation	64	81	
	Radiation	20	24	1
History of neoadjuvant treatment	No neoadjuvant	160	215	
Therapy_type	Ancillary:Chemotherapy	19	13	
	Chemotherapy	59	69	
	Chemotherapy:	1		
	Chemotherapy:Targeted Molecular therapy	1		
	Chemotherapy:Other. specify in notes		1	0.252

Note: *p*-values are calculated using the chi-square test or *t*-test. Some categories are combined to meet statistical requirements.

**Table 4:** Multivariate Cox Regression Coefficients for the 10-Gene Prognostic Model.

Gene Symbol	Coefficient (β)	Hazard Ratio (HR)	HR 95% CI	*p*-Value
*AKR1B1*	0.391	1.478	1.12–1.95	<0.001
*HK2*	−0.180	0.835	0.72–0.97	0.018
*FABP4*	−0.046	0.955	0.92–0.99	0.008
*SERPINE1*	0.068	1.070	1.02–1.12	0.004
*NT5E*	0.242	1.274	1.08–1.50	0.003
*SLC2A3*	0.295	1.343	1.15–1.57	<0.001
*SLC2A2*	0.490	1.632	1.30–2.05	<0.001
*SLC2A1*	0.259	1.296	1.10–1.53	0.002
*NRP1*	0.001	1.001	0.99–1.01	0.890
*SLC16A7*	−0.194	0.824	0.73–0.93	0.002

Note: Coefficients are derived from the multivariate Cox regression model (G1 + G2 training set). HR > 1 indicates a high risk, while HR < 1 indicates a protective factor.

**Table 5:** Predictive Performance of the 10-Gene Model in the Internal Validation Cohort.

Cohort	Data Type	Sample Size	Number at Risk (1/3/5 Years)	Number of Events (1/3/5 Years)	C-Index (SE)	1-Year AUC	3-Year AUC	5-Year AUC
G1	RNA-seq (TCGA)	160	63/14/4	2010/1/1	0.712 (0.065)	0.772	0.842	0.874
G2	RNA-seq (TCGA)	215	87/19/6	11/1/0	0.738 (0.071)	0.768	0.709	0.618 *

Note: The C-index and AUC values are presented with their standard errors (SE) or 95% confidence intervals. * The 5-year AUC for the G2 cohort should be interpreted with extreme caution due to the very small number of patients remaining at risk at this time point (n ≤ 6).

**Table 6:** Predictive Performance of the 10-Gene Model in the External Validation Cohorts.

Cohort	Platform	Sample Size	C-Index (SE)	1-Year AUC	3-Year AUC	5-Year AUC	Log-Rank *p*
ACRG (GSE62254)	GPL570	300	0.503 (0.025)	0.568	0.489	0.481	>0.05
ACRG (z-score)	GPL570	300	0.551 (0.025)	0.585	0.545	0.574	–
GSE26253	GPL570	432	0.603 (0.021)	0.557	0.626	0.603	<0.001

Note: The ACRG cohort utilized the Affymetrix GPL570 chip platform. The event number represents the number of deaths at each time point. The standardized C-index remains close to 0.5, indicating that the model has no clinical predictive value.

**Table 7:** Univariate Cox Regression Analysis of the 10 Genes in the ACRG Cohort.

Gene	HR	HR 95% CI	*p*-Value	Coefficient Sign	Direction Consistency
*AKR1B1*	0.97	0.67–1.41	0.872	+0.391	Inconsistent
*HK2*	0.14	0.03–0.71	0.018	−0.180	Consistent
*FABP4*	2.64	1.51–4.61	0.001	−0.046	Inconsistent
*SERPINE1*	1.95	1.05–3.62	0.036	+0.068	Consistent
*NT5E*	1.25	0.66–2.37	0.493	+0.242	Consistent
*SLC2A3*	1.42	0.87–2.32	0.161	+0.295	Consistent
*SLC2A2*	1.45	0.76–2.76	0.262	+0.490	Consistent
*SLC2A1*	11.64	1.27–106.3	0.029	+0.259	Consistent
*NRP1*	7.05	2.00–24.9	0.002	+0.001	Consistent
*SLC16A7*	2.37	1.44–3.90	0.001	−0.194	Inconsistent

Note: Directional consistency refers to whether the direction of the hazard ratio (HR) is the same as the sign of the model coefficients (a positive coefficient corresponds to HR > 1). The opposing directions of the three genes led to the failure of the composite score.

**Figure A2:**
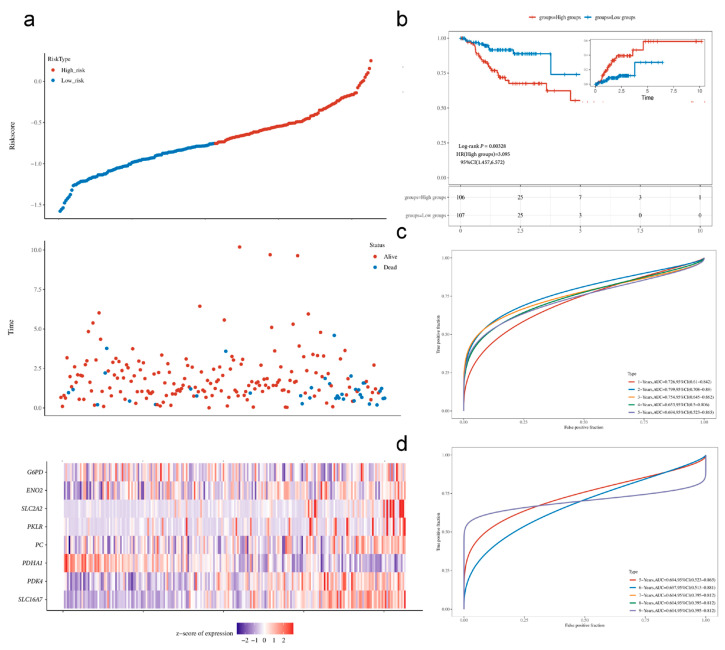
Construction and assessment of the 39-gene prognostic model: (**a**) Distribution of risk scores, patient survival status, and gene expression heatmap for the 39-gene model in the training cohort. Patients are divided into high-risk group (red) and low-risk group (blue) based on the median risk score; (**b**) KM survival curves for high-risk and low-risk groups; (**c**) Time-dependent ROC curve for predicting OS at 1–5 years; (**d**) Time-dependent ROC curve for predicting OS at 5–9 years, with limited sample size after 5 years, necessitating cautious interpretation of the results. Note: In subfigure (**d**), the orange and green curves overlap with the purple curve and are therefore not distinctly visible; only the purple curve is presented.

## Data Availability

The original data presented in this study are openly available in public repositories. The gastric cancer RNA-seq and clinical data from The Cancer Genome Atlas (TCGA) can be accessed via the GDC data portal (https://portal.gdc.cancer.gov/ accessed on 10 February 2026). The microarray datasets GSE62254 (ACRG cohort) and GSE26253, as well as the single-cell datasets GSE163558 and GSE134520, are available from the Gene Expression Omnibus (GEO) database (https://www.ncbi.nlm.nih.gov/geo/ accessed on 10 February 2026). All data were analyzed using publicly available resources as described in the Section 2.

## Supplementary Materials

The following supporting information can be downloaded at: https://www.mdpi.com/article/10.3390/cimb48050477/s1.

## Abbreviations

The following abbreviations are used in this manuscript:

GC	Gastric cancer
LMRGs	lactate metabolism-related genes
TCGA	The Cancer Genome Atlas
PPI	protein–protein interaction
TPM	Transcripts Per Million
DEGs	differentially expressed genes
GO	Gene Ontology
OS	overall survival
HR	hazard ratios
CI	confidence intervals
PCA	principal component analysis
t-SNE	t-distributed stochastic neighbor embedding
TMB	tumor mutation burden
CDF	cumulative distribution function
pDC	plasmacytoid dendritic cells
NK	natural killer
CAFs	cancer-associated fibroblasts
KM	Kaplan–Meier
DOI	digital object identifier
ACRG	Asian Cancer Research Group

## Appendix A

### Independent Prognostic Value of Hub Genes

Univariate Cox regression analysis indicated that, except for *NRP1*, the other 10 hub genes were significantly associated with OS (*p* < 0.05) (Figure A1a, Table A1). Multivariate Cox regression analysis revealed that *AKR1B1*, *SERPINE1*, *NT5E*, *SLC2A3*, *SLC2A2*, and *SLC2A1* were independent risk factors (HR > 1), while *HK2*, *FABP4*, and *SLC16A7* were independent protective factors (HR < 1) (Figure A1b, Table A1). The nomogram constructed based on the multivariate Cox regression results (Figure A1c) can predict the total survival rates of patients at 1, 2, and 3 years, and the calibration curve shows that the predicted probabilities of the model are consistent with the actual observed probabilities (Figure A1d).

**Table A1:** Univariate and multivariate Cox regression analysis of the 11 hub genes.

Gene	Univariate HR (95% CI)	Univariate *p*-Value	Multivariate HR (95% CI)	Multivariate *p*-Value
*AKR1B1*	1.39 (1.16–1.67)	<0.001	1.32 (1.07–1.64)	0.011
*HK2*	1.01 (0.88–1.15)	0.928	0.96 (0.81–1.14)	0.666
*FABP4*	1.11 (1.01–1.21)	0.029	1.03 (0.92–1.15)	0.639
*PDK4*	1.10 (1.00–1.20)	0.039	1.03 (0.91–1.17)	0.602
*SERPINE1*	1.26 (1.13–1.40)	<0.001	1.21 (1.04–1.40)	0.015
*NT5E*	1.25 (1.09–1.43)	0.001	1.19 (1.00–1.40)	0.044
*SLC2A3*	1.20 (1.04–1.39)	0.014	0.91 (0.72–1.15)	0.437
*SLC2A2*	1.17 (0.97–1.41)	0.108	1.22 (0.99–1.51)	0.064
*SLC2A1*	1.03 (0.91–1.17)	0.608	1.02 (0.87–1.21)	0.770
*NRP1*	1.44 (1.18–1.76)	<0.001	1.03 (0.74–1.43)	0.875
*SLC16A7*	1.17 (0.99–1.39)	0.065	0.92 (0.72–1.17)	0.473

## Appendix B. Construction and Validation of the 39-Gene Prognostic Model

The distribution of risk scores for the 39-gene model, patient survival status, and gene expression heatmap can be found in Figure A2. The KM curve shows that the high-risk group has a significantly poorer prognosis (log-rank *p* < 0.001, Figure A2). The time-dependent ROC curves indicate AUC values ranging from 0.656 to 0.705 for 1 to 5 years (Figure A2), while the AUC for 5 to 9 years declines due to the reduced sample size (Figure A2). The risk coefficients are as follows:

*Riskscore* = (0.5157) × *LDHA* + (0.0213) × *SLC16A1* + (0.0893) × *SLC16A3* + (0.1772) × *SLC16A7* + (−0.3305) × *SLC16A8* + (−0.1094) × *PDK1* + (−0.0283) × *PDK2* + (0.1825) × *PDK3* + (0.1904) × *PDK4* + (0.0411) × *PDHA1* + (0.0979) × *PDHB* + (−0.5271) × *DLAT* + (0.0757) × *DLD* + (0.1002) × *PDP1* + (0.3606) × *PDP2* + (0.1442) × *PC* + (0.1135) × *PKM* + (−0.1499) × *PKLR* + (0.0063) × *HK1* + (−0.0183) × *HK2* + (−0.2621) × *HK3* + (−0.061) × *SLC2A1* + (−0.0123) × *SLC2A2* + (0.3372) × *SLC2A3* + (−0.2038) × *SLC2A4* + (0.1035) × *GAPDH* + (−0.3885) × *PGK1* + (−0.2177) × *PGAM1* + (0.0608) × *ENO1* + (−0.3034) × *ENO2* + (0.3514) × *ENO3* + (−0.0029) × *ALDOA* + (0.1985) × *ALDOC* + (−0.1152) × *TPI1* + (0.0621) × *PFKP* + (0.1726) × *PFKL* + (0.1683) × *PFKM* + (−0.0287) × *G6PD* + (−0.3064) × *MCTP2* (A1)

Although the 39-gene model had a slightly higher AUC than the 10-gene model in the training set (1–5 years AUC: 0.656–0.705 vs. 0.719–0.864), it includes more genes, which poses a risk of overfitting. Additionally, the C-index in the internal validation cohorts G1 and G2 was slightly lower than that of the 10-gene model (Table A2). Furthermore, Landmark analysis shows that the C-index of the 10-gene model at the 3-year landmark point significantly outperformed the 39-gene model (G1: 0.769 vs. 0.462; G2: 0.818 vs. 0.545) (Table A2), indicating that the 10-gene model has an advantage in predicting short-term survival. Considering the simplicity, stability, and biological interpretability of the models, we ultimately selected the 10-gene model as the core model for subsequent analyses.

**Table A2:** Landmark analysis results for the 39-gene model and 10-gene model in cohorts G1 and G2 (3 years).

Cohort	Model	Landmark (Years)	Remaining Sample Size	Number of Events	C-Index (SE)	1-Year AUC	2-Year AUC	3-Year AUC
G1	39-gene	3	14	2	0.462 (0.283)	0.143	0.500	0.500
G1	10-gene	3	14	2	0.769 (0.149)	0.571	0.875	1.000
G2	39-gene	3	19	1	0.545 (0.150)	0.714	0.667	1.000
G2	10-gene	3	19	1	0.818 (0.116)	1.000	1.000	1.000

Note: The 5-year landmark analysis could not be calculated due to zero event counts in the samples; therefore, it is not listed.

**Table A3:** Summary and interpretation of C-index results.

Cohort	Model	C-Index	Standard Error	95% CI (Approximate)
G1	10 Genes	0.712	0.065	0.585–0.839
G1	39 Genes	0.653	0.089	0.479–0.827
G2	10 Genes	0.738	0.071	0.599–0.877
G2	39 Genes	0.795	0.058	0.681–0.909

## Appendix C. External Validation in the ACRG Cohort (GSE62254)

In the ACRG cohort (GSE62254), the C-index for risk scores calculated using the original model coefficients was only 0.503 (SE = 0.025), with time-dependent AUCs of 0.568, 0.489, and 0.481 for 1, 3, and 5 years, respectively (Table 7), indicating that the model completely lost its predictive ability in this cohort. After z-score normalization, the C-index slightly improved to 0.551 (SE = 0.025), with AUCs of 0.585, 0.545, and 0.574, but it still held no clinical value (Table 6). To explore the reasons for this failure, we analyzed the risk scores across the ACRG molecular subtypes (EMT, MSI, TP53 negative, and TP53 positive), and there were no significant differences found (ANOVA *p* = 0.469, Table A4), suggesting that the model failed to capture biologically meaningful variations in this cohort.

**Table A4:** Association analysis of risk scores and ACRG molecular subtypes.

ACRG Molecular Subtype	Sample Size	Mean Risk Score	Standard Deviation	ANOVA *p*-Value
EMT	46	2.41	4.19	
MSI	68	1.72	4.26	
TP53neg	107	2.48	3.87	
TP53positive	79	2.75	3.91	
Total	300	2.37	4.02	0.469

Note: The *p*-values are from one-way analysis of variance (ANOVA), indicating that there are no significant differences in risk scores among the four ACRG molecular subtypes.
