# Supplementary material for: Cellular Origins and Context-Dependent Prognostic Effects of Lactate Metabolism Genes Reveal Novel Molecular Subtypes in Gastric Cancer
Source: Curr Issues Mol Biol. 2026 May 4;48(5):477. doi: 10.3390/cimb48050477 (PMC13204596; doi:10.3390/cimb48050477)
Supplement: Supplementary file 1 [file cimb-48-00477-s001.zip › Supplementary Materials.pdf]

Supplementary Materials

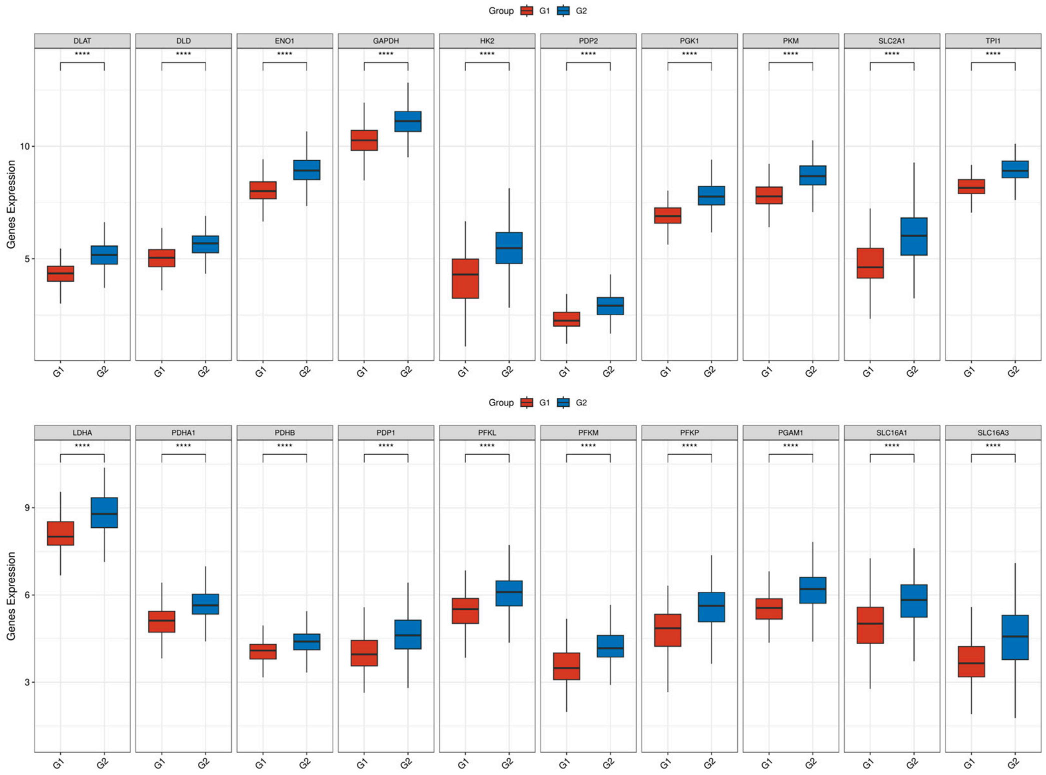

Figure S1. Box plots of the expression levels of 49 LMRGs in G1 and G2 subtypes: The box plots display the expression levels of 49 genes in the G1 and G2 subtypes, with P-values calculated using the Wilcoxon test.

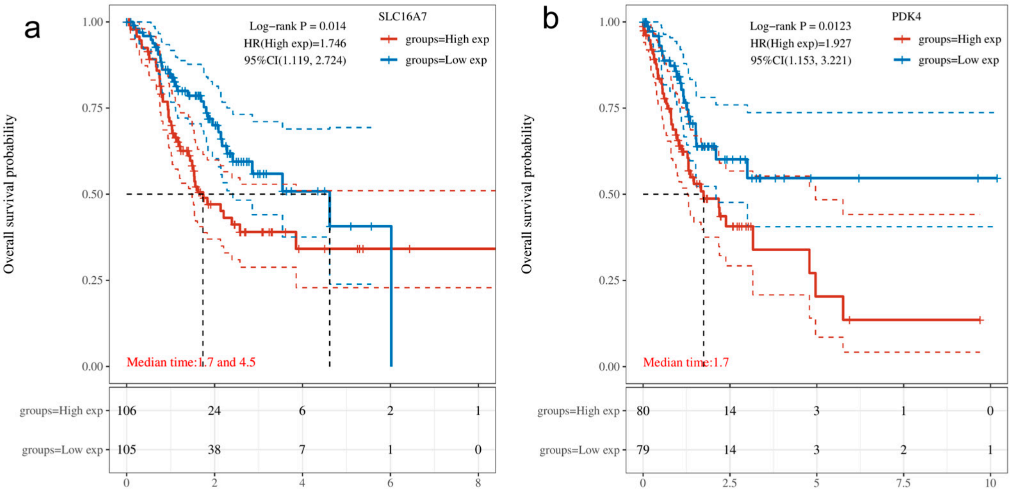

Figure S2. Survival analysis of 49 LMRGs: (**a**)Univariate Cox regression forest plot for the 49 genes in the G1 cohort (showing only genes with P < 0.05);(**b**)Univariate Cox regression forest plot for the 49 genes in the G2 cohort.

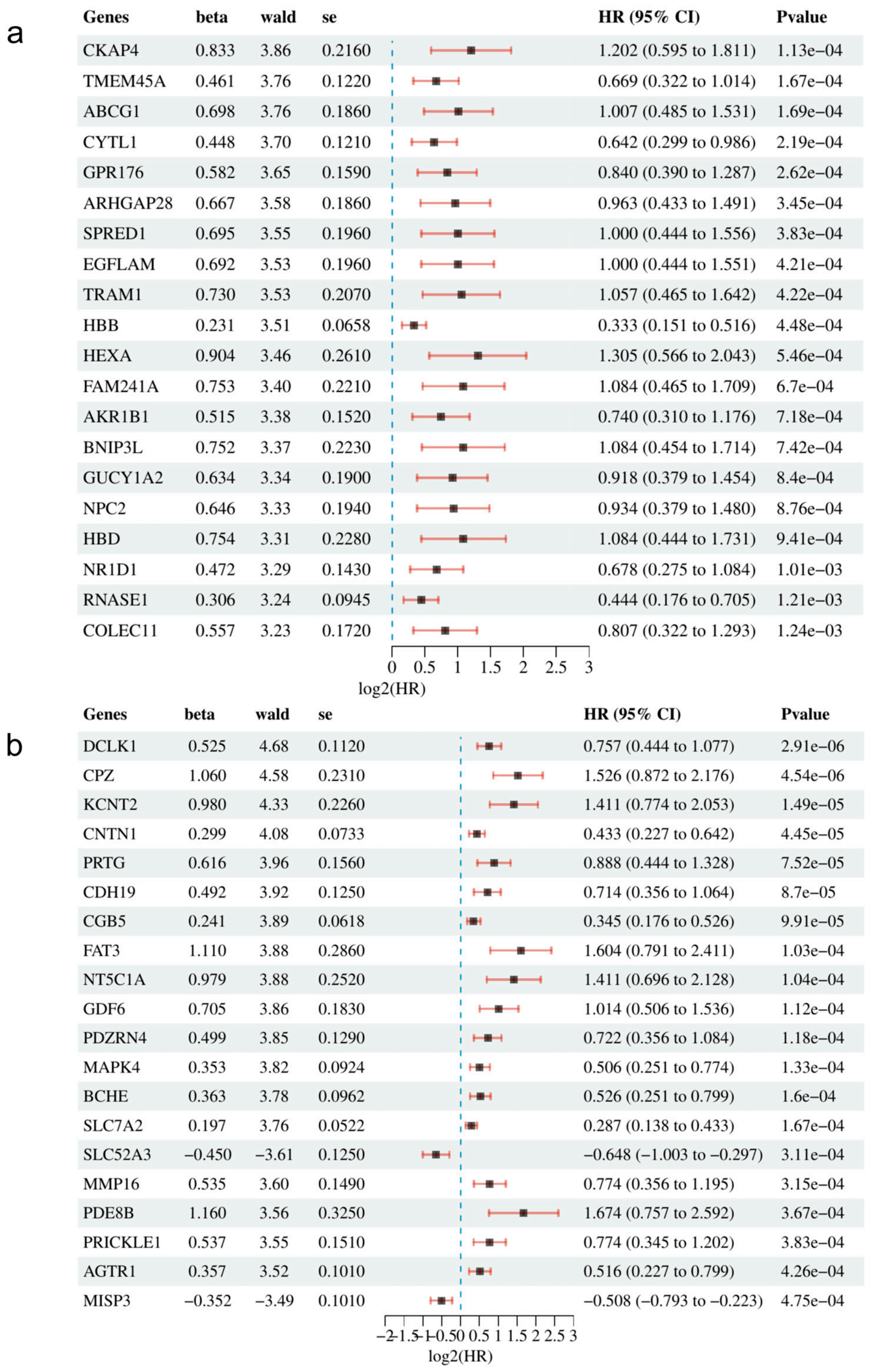

a

Genes	beta	wald	se	HR (95% CI)	Pvalue
CKAP4	0.833	3.86	0.2160	1.202 (0.595 to 1.811)	1.13e−04
TMEM45A	0.461	3.76	0.1220	0.669 (0.322 to 1.014)	1.67e−04
ABCG1	0.698	3.76	0.1860	1.007 (0.485 to 1.531)	1.69e−04
CYTL1	0.448	3.70	0.1210	0.642 (0.299 to 0.986)	2.19e−04
GPR176	0.582	3.65	0.1590	0.840 (0.390 to 1.287)	2.62e−04
ARHGAP28	0.667	3.58	0.1860	0.963 (0.433 to 1.491)	3.45e−04
SPRED1	0.695	3.55	0.1960	1.000 (0.444 to 1.556)	3.83e−04
EGFLAM	0.692	3.53	0.1960	1.000 (0.444 to 1.551)	4.21e−04
TRAM1	0.730	3.53	0.2070	1.057 (0.465 to 1.642)	4.22e−04
HBB	0.231	3.51	0.0658	0.333 (0.151 to 0.516)	4.48e−04
HEXA	0.904	3.46	0.2610	1.305 (0.566 to 2.043)	5.46e−04
FAM241A	0.753	3.40	0.2210	1.084 (0.465 to 1.709)	6.7e−04
AKR1B1	0.515	3.38	0.1520	0.740 (0.310 to 1.176)	7.18e−04
BNIP3L	0.752	3.37	0.2230	1.084 (0.454 to 1.714)	7.42e−04
GUCY1A2	0.634	3.34	0.1900	0.918 (0.379 to 1.454)	8.4e−04
NPC2	0.646	3.33	0.1940	0.934 (0.379 to 1.480)	8.76e−04
HBD	0.754	3.31	0.2280	1.084 (0.444 to 1.731)	9.41e−04
NR1D1	0.472	3.29	0.1430	0.678 (0.275 to 1.084)	1.01e−03
RNASE1	0.306	3.24	0.0945	0.444 (0.176 to 0.705)	1.21e−03
COLEC11	0.557	3.23	0.1720	0.807 (0.322 to 1.293)	1.24e−03

b

Genes	beta	wald	se	HR (95% CI)	Pvalue
DCLK1	0.525	4.68	0.1120	0.757 (0.444 to 1.077)	2.91e−06
CPZ	1.060	4.58	0.2310	1.526 (0.872 to 2.176)	4.54e−06
KCNT2	0.980	4.33	0.2260	1.411 (0.774 to 2.053)	1.49e−05
CNTN1	0.299	4.08	0.0733	0.433 (0.227 to 0.642)	4.45e−05
PRTG	0.616	3.96	0.1560	0.888 (0.444 to 1.328)	7.52e−05
CDH19	0.492	3.92	0.1250	0.714 (0.356 to 1.064)	8.7e−05
CGB5	0.241	3.89	0.0618	0.345 (0.176 to 0.526)	9.91e−05
FAT3	1.110	3.88	0.2860	1.604 (0.791 to 2.411)	1.03e−04
NT5C1A	0.979	3.88	0.2520	1.411 (0.696 to 2.128)	1.04e−04
GDF6	0.705	3.86	0.1830	1.014 (0.506 to 1.536)	1.12e−04
PDZRN4	0.499	3.85	0.1290	0.722 (0.356 to 1.084)	1.18e−04
MAPK4	0.353	3.82	0.0924	0.506 (0.251 to 0.774)	1.33e−04
BCHE	0.363	3.78	0.0962	0.526 (0.251 to 0.799)	1.6e−04
SLC7A2	0.197	3.76	0.0522	0.287 (0.138 to 0.433)	1.67e−04
SLC52A3	−0.450	−3.61	0.1250	−0.648 (−1.003 to −0.297)	3.11e−04
MMP16	0.535	3.60	0.1490	0.774 (0.356 to 1.195)	3.15e−04
PDE8B	1.160	3.56	0.3250	1.674 (0.757 to 2.592)	3.67e−04
PRICKLE1	0.537	3.55	0.1510	0.774 (0.345 to 1.202)	3.83e−04
AGTR1	0.357	3.52	0.1010	0.516 (0.227 to 0.799)	4.26e−04
MISP3	−0.352	−3.49	0.1010	−0.508 (−0.793 to −0.223)	4.75e−04

Figure S3. Genome-wide bulk survival analysis: (**a**)Volcano plot of the genome-wide univariate Cox regression in the G1 cohort;(**b**)Volcano plot of the genome-wide univariate Cox regression in the G2 cohort.

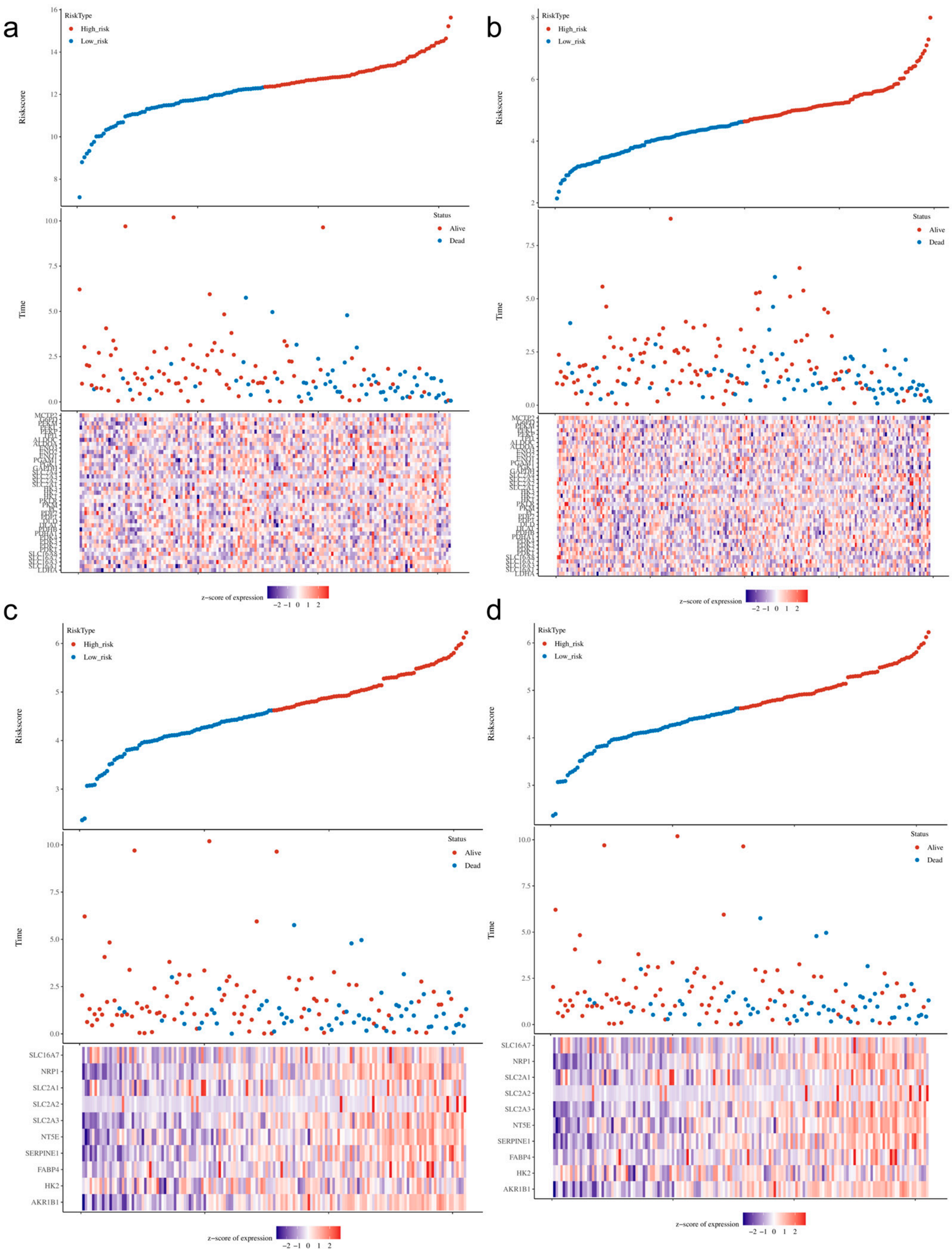

Figure S4. Risk score distribution and model performance in the internal validation cohort: Risk score distribution of the 39-gene model in G1 (a) and G2 (b), along with patient survival status and gene expression heatmap; risk score distribution of the 10-gene model in G1 (c) and G2 (d), along with patient survival status and gene expression heatmap.

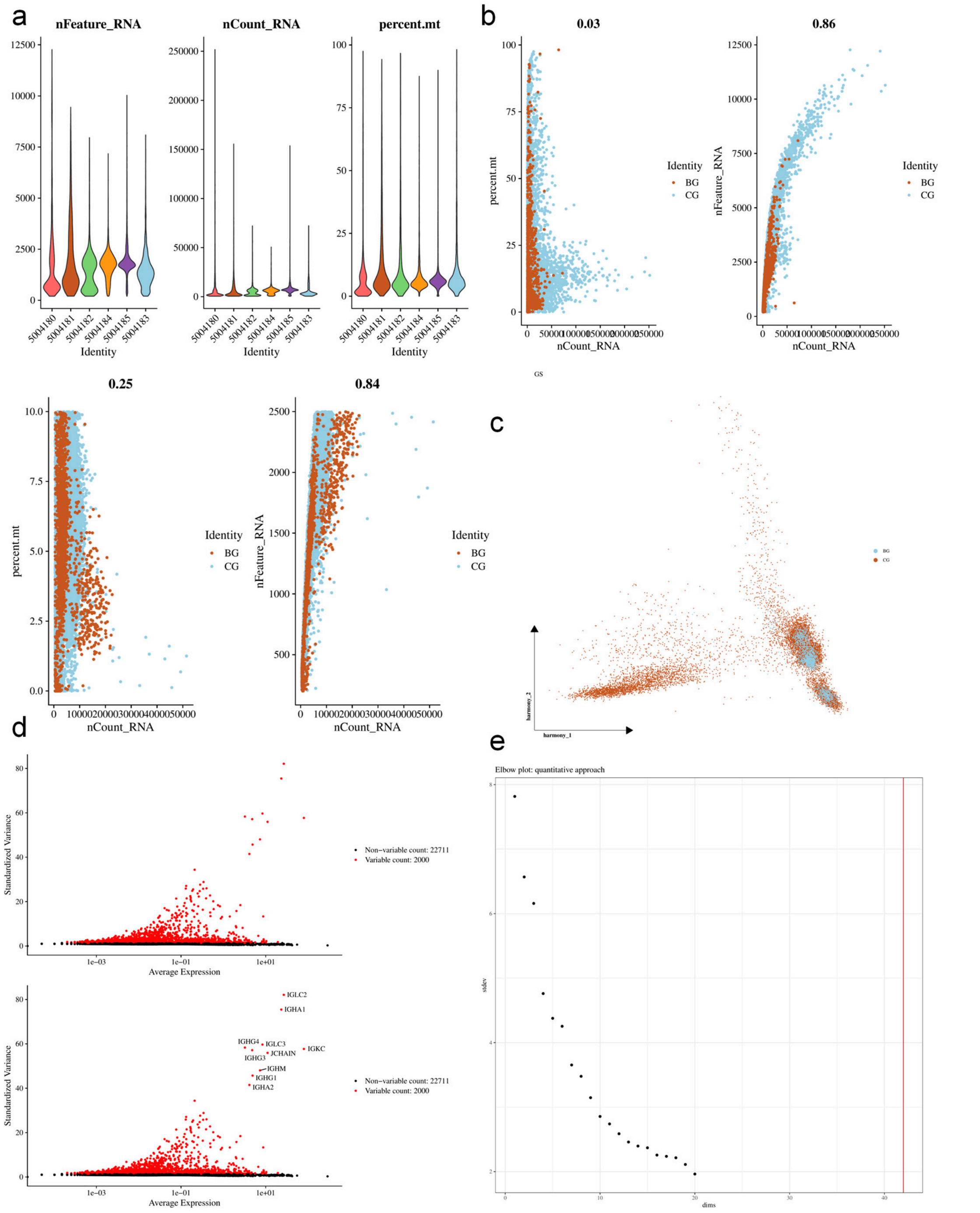

Figure S5. Comprehensive quality control and data preprocessing workflow for the GSE163558 single-cell RNA-seq dataset. (A) Violin plots displaying the distribution of quality control (QC) metrics across all cells before filtering: the number of detected genes per cell (nFeature_RNA), the total number of unique molecular identifiers per cell (nCount_RNA), and the percentage of mitochondrial gene content (percent.mt). The dashed red lines indicate the filtering thresholds applied (nFeature_RNA: 200 – 6,500; nCount_RNA < 50,000; percent.mt < 10%). (B) Boxplots illustrating the distribution of the number of detected genes (left panel) and mitochondrial gene percentage (right panel) across the six individual samples (GSM5004180 – GSM5004185)

after applying the QC filters. The consistent distributions across samples support the absence of gross technical outliers. (C) Principal component analysis (PCA) plot of the integrated dataset colored by sample-of-origin. The substantial overlap of cells from different samples within the PC1 – PC2 space indicates that technical batch effects are minimal and that biological variation is the primary driver of clustering. (D) Scatter plot identifying the top 2,000 highly variable genes (HVGs) used for downstream dimensionality reduction. Each point represents a single gene. The top 10 most variable genes are highlighted in red and labeled with their gene symbols. (E) Elbow plot depicting the standard deviation of each principal component (PC). The red dashed line marks the "elbow" point, after which the incremental variance explained by additional PCs diminishes. Based on this heuristic, the first 18 PCs were selected for t-SNE clustering and subsequent analyses.

Table S1. AUC values and the number of at-risk individuals over time in the G1 and G2 cohorts.

Time (years)	G1 10-gene AUC (95% CI)	G1 39-gene AUC (95% CI)	G1 Number at risk	G2 10-gene AUC (95% CI)	G2 39-gene AUC (95% CI)	G2 Number at risk
1	0.736 (0.660–0.812)	0.772 (0.692–0.853)	63	0.619 (0.537–0.702)	0.768 (0.685–0.850)	87
2	0.688 (0.594–0.781)	0.852 (0.786–0.917)	30	0.692 (0.616–0.769)	0.711 (0.632–0.789)	41
3	0.790 (0.693–0.887)	0.842 (0.764–0.919)	14	0.677 (0.583–0.771)	0.709 (0.625–0.792)	19
4	0.786 (0.666–0.905)	0.834 (0.735–0.933)	7	0.744 (0.633–0.855)	0.614 (0.505–0.723)	7
5	0.844 (0.743–0.944)	0.874 (0.772–0.976)	4	0.699 (0.554–0.844)	0.618 (0.497–0.740)	6
6	0.904 (0.815–0.993)	0.860 (0.701–1.018)	4	0.503 (0.304–0.702)	0.587 (0.427–0.748)	2
7	0.872 (0.771–0.974)	0.813 (0.621–1.005)	4	-(Insufficient follow-up)	-(Insufficient follow-up)	0
8	0.872 (0.771–0.974)	0.813 (0.621–1.005)	4	-(Insufficient follow-up)	-(Insufficient follow-up)	0
9	0.872 (0.771–0.974)	0.813 (0.621–1.005)	4	-(Insufficient follow-up)	-(Insufficient follow-up)	0

Note: There were no at-risk individuals in the G2 cohort after 6 years, so the AUC value is not listed. The AUC values for the G1 cohort from years 7 to 9 are the same, and their CI are also identical.

Table S2. Correlation between the 10-gene model and immune cell infiltration.

Algorithm	Immune Cell Type	Spearman Correlation Coefficient	P-Value
CIBERSORT	B cell naive	0.1129	0.1011
	B cell memory	0.1263	0.0664
	B cell plasma	0.0537	0.4367
	T cell CD8+	0.1356	0.0487 *
	T cell CD4+ memory resting	-0.0387	0.5748
	T cell CD4+ memory activated	-0.1943	0.0045 **
	T cell follicular helper	-0.1262	0.0666
	T cell regulatory (Tregs)	0.0774	0.2617

	T cell gamma delta	0.0091	0.8954
	NK cell resting	-0.1322	0.0547
	NK cell activated	0.0248	0.7197
	Monocyte	0.2809	3.33e-05 **
	Macrophage M0	-0.2193	0.0013 **
	Macrophage M1	-0.0786	0.2546
	Macrophage M2	0.1942	0.0046 **
	Myeloid dendritic cell resting	0.1271	0.0647
	Myeloid dendritic cell activated	-0.0014	0.9841
	Mast cell activated	0.2143	0.0017 **
	Mast cell resting	-0.1672	0.0148 *
	Eosinophil	-0.0445	0.5191
	Neutrophil	-0.2234	0.0011 **
EPIC	B cell naive	0.1129	0.1011
	B cell memory	0.1263	0.0664
	B cell plasma	0.0537	0.4367
	T cell CD8+	0.1356	0.0487 *
	T cell CD4+ memory resting	-0.0387	0.5748
	T cell CD4+ memory activated	-0.1943	0.0045 **
	T cell follicular helper	-0.1262	0.0666
	T cell regulatory (Tregs)	0.0774	0.2617
	T cell gamma delta	0.0091	0.8954
	NK cell resting	-0.1322	0.0547
	NK cell activated	0.0248	0.7197
	Monocyte	0.2809	3.33e-05 **
	Macrophage M0	-0.2193	0.0013 **
	Macrophage M1	-0.0786	0.2546
	Macrophage M2	0.1942	0.0046 **
	Myeloid dendritic cell resting	0.1271	0.0647
	Myeloid dendritic cell activated	-0.0014	0.9841
	Mast cell activated	0.2143	0.0017 **
	Mast cell resting	-0.1672	0.0148 *
	Eosinophil	-0.0445	0.5191
	Neutrophil	-0.2234	0.0011 **
MCP-counter	T cell	0.1663	0.0155 *
	T cell CD8+	0.1722	0.0121 *
	cytotoxicity score	0.0224	0.7459
	NK cell	0.0968	0.1602
	B cell	0.3254	1.47e-06 **
	Monocyte	0.2862	2.51e-05 **
	Macrophage/Monocyte	0.2862	2.51e-05 **
	Myeloid dendritic cell	0.3882	6.59e-09 **
	Neutrophil	0.1160	0.0920
	Endothelial cell	0.3912	4.94e-09 **
TIMER	B cell	0.2575	0.00015 **
	T cell CD4+	0.3867	7.63e-09 **

	T cell CD8+	0.1898	0.0056 **
	Neutrophil	0.2081	0.0024 **
	Macrophage	0.5129	1.28e-15 **
	Myeloid dendritic cell	0.2764	4.81e-05 **
xCELL	Myeloid dendritic cell activated	0.2633	0.00010 **
	B cell	0.0880	0.2021
	T cell CD4+ memory	-0.1453	0.0345 *
	T cell CD4+ naive	0.1478	0.0314 *
	T cell CD4+ (non-regulatory)	-0.0411	0.5522
	T cell CD4+ central memory	-0.0090	0.8964
	T cell CD4+ effector memory	-0.1156	0.0932
	T cell CD8+ naive	-0.1444	0.0356 *
	T cell CD8+	0.0758	0.2720
	T cell CD8+ central memory	0.0803	0.2444
	T cell CD8+ effector memory	-0.0443	0.5215
	Class-switched memory B cell	0.0102	0.8825
	Common lymphoid progenitor	-0.3061	5.63e-06 **
	Common myeloid progenitor	0.0347	0.6156
	Myeloid dendritic cell	0.2419	0.00038 **
	Endothelial cell	0.3464	2.29e-07 **
	Eosinophil	0.2385	0.00046 **
	Granulocyte-monocyte progenitor	0.0766	0.2670
	Hematopoietic stem cell	0.4343	3.65e-11 **
	Macrophage	0.0728	0.2916
	Macrophage M1	0.0670	0.3314
	Macrophage M2	0.1664	0.0153 *
	Mast cell	-0.0250	0.7179
	B cell memory	0.0989	0.1514
	Monocyte	0.1218	0.0767
	B cell naive	0.1189	0.0842
	Neutrophil	-0.0291	0.6739
	NK cell	-0.1379	0.0448 *
	T cell NK	0.0709	0.3045
	Plasmacytoid dendritic cell	-0.1570	0.0222 *
	B cell plasma	-0.0876	0.2040
	T cell gamma delta	-0.1723	0.0120 *
	T cell CD4+ Th1	-0.2857	2.40e-05 **
	T cell CD4+ Th2	-0.2224	0.0011 **
	T cell regulatory (Tregs)	-0.0926	0.1790
	immune score	0.1494	0.0297 *
	stroma score	0.4312	5.20e-11 **
	microenvironment score	0.3580	1.02e-07 **

Note: * P < 0.05, ** P < 0.01. A P-value of 0.0000 indicates it is less than 1e-15. The results from CIBERSORT and EPIC are consistent due to the same original data.

Table S3. Test of the proportional hazards (PH) assumption for the 10-gene multivariate Cox model using Schoenfeld residuals.

Gene	Chisq	P-value
AKR1B1	0.11	0.740
HK2	0.27	0.603
FABP4	4.14	0.042
SERPINE1	0.03	0.862
NT5E	2.01	0.156
SLC2A3	0.85	0.357
SLC2A2	0.54	0.462
SLC2A1	1.23	0.267
NRP1	0.01	0.920
SLC16A7	1.58	0.209
GLOBAL	14.33	0.130

Note: A non-significant p-value ($P > 0.05$) indicates that the PH assumption is not violated. The global test P-value of 0.130 confirms the overall validity of the model. The marginal P-value for FABP4 (0.042) suggests a minor time-dependent variation in its hazard ratio, warranting cautious interpretation of its precise coefficient.

Table S4. Top 10 marker genes for each cell cluster identified in the GSE163558 sin-gle-cell RNA-seq dataset.

	p_val	avg_log2FC	pct.1	pct.2	p_val_adj	cluster	gene
KIT	0	9.90629619222938	0.93	0.003	0	18	KIT
SOD3	0	9.89072395403766	0.289	0.001	0	14	SOD3
HDC	0	9.76253716848896	0.649	0.002	0	18	HDC
MGP	0	9.72154637942603	0.635	0.009	0	14	MGP
COL14A1	0	9.69612298992802	0.269	0.001	0	14	COL14A1
RAMP2	0	9.64832567144886	0.285	0.001	0	14	RAMP2
IGHA1	0	9.54733971161177	0.841	0.162	0	10	IGHA1
CADPS	0	9.49589421259811	0.333	0	0	18	CADPS
JCHAIN	0	9.36616950068683	0.97	0.102	0	10	JCHAIN
IGHA2	0	9.18380882043818	0.651	0.053	0	10	IGHA2

Table S5. Spearman correlation analysis between FABP4 expression and gene signature scores in the G2 subtype.

Gene Signature	Component Genes	Spearman ρ	95% CI	P-value
Proliferative CD8+ T cell signature	MKI67,CD3D,CD8A,GZMB	0.19	0.06–0.32	0.005
Cancer-associated fibroblast (CAF) signature	ACTA2,FAP,COL1A1,PDGFRA	0.48	0.37–0.58	< 0.001

Note: Signature scores were calculated using single-sample Gene Set Enrichment Analysis (ssGSEA). Correlation coeffi-cients and P-values were computed using Spearman's rank correlation test. The stronger correlation with the CAF signature supports the hypothesis that in the G2 subtype, bulk FABP4 expression is predominantly driven by stromal fibroblasts rather than proliferative T cells.

Table S6. Bootstrap internal validation of the 10-gene prognostic model.

Metric	Value
Number of bootstrap resamples	1,000
Apparent C-index (training data)	0.738
Test C-index (out-of-bag samples)	0.723
Optimism	0.017
Optimism-corrected C-index	0.721
Cross-validated C-index (fromcv.glmnet)	0.698

Note: Bootstrap internal validation was performed using the validate function from the rms R package with 1,000 resamples. Apparent C-index repre-sents model performance on the full training dataset. Test C-index is the average performance on out-of-bag samples not included in each boot-strap resample. Optimism is the difference between apparent and test C-indices. Optimism-corrected C-index = apparent C-index − optimism. The close agreement between the optimism-corrected C-index (0.721), cross-validated C-index (0.698), and apparent C-index (0.738) indicates that the model's performance is stable and that overfitting is minimal.

Table S7. Stability assessment of the 10-gene prognostic model via repeated 5-fold cross-validation (100 iterations, 500 validation folds) within the TCGA training cohort.

Statistic	C-index Value
Median	0.71
Interquartile range (IQR)	0.67 – 0.75
Minimum	0.59
Maximum	0.82
Apparent C-index (full TCGA cohort)	0.738
Cross-validated C-index (fromcv.glmnet)	0.698

Note: In each of the 100 iterations, the TCGA cohort was randomly split into five folds. The LASSO Cox model was trained on four folds and validated on the held-out fold, producing a C-index for each validation fold. The distribution of these 500 C-index values characterizes the model's per-formance variability due to random sampling.

Table S8. Sensitivity analysis: performance of the 9-gene model (FABP4 excluded) in the ACRG external validation cohort (GSE62254, n=300).

Metric	9-gene model (FABP4 excluded)	Original 10-gene model
C-index (95% CI)	0.51 (0.46–0.56)	0.503 (0.454–0.552)
1-year AUC	0.57	0.568
3-year AUC	0.49	0.489
5-year AUC	0.48	0.481
Log-rank P-value	> 0.05	> 0.05

Note: The 9-gene model was constructed by removing FABP4 from the initial 11 hub genes and repeating the entire LASSO Cox regression pipeline (10-fold cross-validation on TCGA, coefficient estimation). Risk scores were calculated in the ACRG cohort using the new coefficients.

The near-identical performance metrics indicate that FABP4 alone does not account for the model's failure in the ACRG cohort.
